# Cultural attitudes toward sport psychology: insights from Italian athletes and coaches

**DOI:** 10.3389/fpsyg.2025.1630005

**Published:** 2025-08-04

**Authors:** Gabriele Simonti, Cindra Kamphoff, Michelle McAlarnen, Panagiotis Kratimenos, Alessandro Quartiroli

**Affiliations:** ^1^College of Allied Health and Nursing, Sports, Exercise, and Performance Psychology, Minnesota State University, Mankato, MN, United States; ^2^Children’s National Hospital, George Washington University School of Medicine and Health Sciences, Washington, DC, United States; ^3^Psychology Department, University of Wisconsin-La Crosse, La Crosse, WI, United States; ^4^School of Psychology, Sport, and Health Science, University of Portsmouth, Portsmouth, United Kingdom

**Keywords:** athletes, coaches, sports psychology, attitudes toward sport psychology, cultural context

## Abstract

**Introduction:**

Sport psychology (SP) examines the mental factors that influence athletic performance and well-being. Although the field has experienced substantial global growth over the past decade, much of the existing literature remains rooted in AngloSaxon cultural contexts, raising questions about its applicability in other cultural contexts.

**Methods:**

This study explored context-specific perceptions of SP by examining the attitudes of two key sport stakeholders—Italian athletes (*n* = 298; 50.17%) and coaches (*n* = 296; 49.83%). Participants (male = 324; 54.45%; female = 270; 45.55%) represented both individual and team sports, across elite and non-elite competitive levels. An online survey assessed participants’ perceptions and attitudes toward SP services and professionals. Using Brislin’s back-translation method, we adapted the SPA-R and SPARC-2 into Italian.

**Results:**

Confirmatory factor analysis confirmed the internal structure of the instruments in the Italian sample. We then examined differences in attitudes by stakeholder role, gender, age, and competitive level. Overall, both athletes and coaches reported low levels of stigma and high confidence in SP and its services. However, significant differences were observed across demographic groups

**Discussion:**

This study provides a culturally grounded snapshot of the current sport psychology landscape in Italy, highlighting context-specific attitudes and offering valuable insights for enhancing the cultural relevance of SP services and advancing global understanding of the field.

## Introduction

Sport psychology (SP) is primarily concerned with addressing the psychological challenges that arise in the context of sport participation (Jarvis, 2006). SP practitioners primarily work with athletes and coaches, the central recipients of SP services, while also supporting teams and sport organizations across both professional and recreational settings, with interventions tailored to specific performance needs (Fortin-Guichard et al., 2018).

Yet, the effectiveness and widespread uptake of SP services often hinge on the attitudes of those they are intended to support (Martin et al., 2012) Early research highlighted a general reluctance among athletes to engage with SP, marked by skepticism and a hesitancy to seek psychological support (Ravizza, 1988). Those who did access such services frequently encountered stigma, experiencing a “negative halo effect” that adversely affected their social standing within the sport environment (Linder et al., 1989).

More recent evidence, however, points to a positive shift in these perceptions. Both athletes and coaches have demonstrated growing acceptance and increased use of SP services (Fortin-Guichard et al., 2018). This evolving attitude is mirrored in the growing institutionalization of SP within sport organizations and competitive contexts (Martin et al., 2002; Martin et al., 2004). By the early 2000s, collegiate athletes were increasingly viewing SP in a more favorable light, largely free from the negative connotations previously associated with seeking psychological support (Gentner et al., 2004; Van Raalte et al., 2016). Similar patterns have been observed among American high school coaches (Zakrajsek et al., 2011) and youth sport coaches in the United Kingdom (Barker and Winter, 2014). Despite this encouraging trend, resistance persists in certain segments of the sports community, and attitudinal discrepancies continue to influence how SP services are integrated and utilized across various settings.

Attitudes toward SP are shaped by a range of demographic and contextual factors, including gender, age, and competitive level. Each dimension contributes uniquely to how SP is perceived and engaged with by athletes and coaches. Among these, gender has emerged as one of the most consistently examined influences (Fortin-Guichard et al., 2018). For example, studies conducted in both the UK and the US have found that female athletes are more likely to seek psychological support and hold positive views of SP. Similarly, female coaches in the US tend to report more supportive attitudes toward SP (Brooks and Bull, 1999; Martin, 2005; Zakrajsek and Zizzi, 2007; Wrisberg et al., 2010). However, not all findings are consistent: for instance, research on US high school coaches found no significant gender-based differences in SP attitudes (Nelson, 2008), highlighting the potential role of cultural and contextual variables.

Age is another key factor. In Western countries such as the US and the UK, younger athletes, particularly those at non-elite levels, are more likely to associate help-seeking with stigma or fear of negative judgment for engaging with SP services (Martin, 2005; Bell et al., 2022). Encouragingly, exposure to SP interventions—such as mental skills training—can positively shift these attitudes, especially among younger athletes (Sharp et al., 2013). In contrast, older and elite-level athletes tend to express more supportive and open attitudes toward SP (Gentner et al., 2004; Wrisberg et al., 2009).

Competitive level also plays a role in shaping perceptions. While coaches generally express positive attitudes toward SP regardless of competitive context, younger coaches (under 30) tend to report more negative views compared to their older peers (Zakrajsek et al., 2011; Michel, 2013). Taken together, these findings emphasize the complex interplay of gender, age, and competitive experience in shaping SP attitudes, and they suggest a need for further research that accounts for these intersecting variables across a wider range of sporting populations.

Beyond individual and contextual dimensions, broader cultural and national frameworks also exert a significant influence on how SP is perceived and adopted. While sport is increasingly understood as a transnational phenomenon—with globalization facilitating the exchange of cultural practices and shaping regional identities (Giulianotti and Robertson, 2007)—the current body of SP research remains largely concentrated in Anglo-Saxon countries (Fortin-Guichard et al., 2018). This geographic concentration limits our understanding of the global diversity in SP attitudes and practices.

Cross-cultural comparisons reveal meaningful differences. Athletes in countries such as Great Britain and New Zealand generally hold more positive views of SP, while those from Eastern cultures tend to exhibit greater skepticism (Sullivan and Hodge, 1991; Byrne and Cusack, 2015; Ong and Harwood, 2018). Coaches in Western nations also demonstrate openness to SP, but those in countries such as the Philippines appear more hesitant (Barker and Winter, 2014; Guinto-Adviento, 2016). Comparative studies among athletes in the US, UK, Spain, and Germany show that those in the UK and Spain are more likely to trust SP consultants and report less stigma around using psychological services (Martin et al., 2004; Checa et al., 2023). These findings reinforce the powerful influence of cultural and national settings and the need for greater geographic diversity in SP research.

As emphasized by Schinke and Stambulova (2017) cultural context significantly shapes the practice of sport psychology, influencing everything from intervention strategies to conceptual frameworks. Yet, existing research tends to generalize across regions, lacking the cultural specificity needed to understand how SP is understood and utilized in different contexts.

Despite Italy’s prominent role in global sport—as evidenced by its significant presence in the Paris 2024 Olympic Games (CONI - Comitato Olimpico Nazionale Italiano, 2024) and the increasing adoption of SP services—there is a notable gap in empirical research examining how Italian athletes and coaches perceive SP. This study seeks to address that gap by investigating attitudes toward SP in the Italian context. By assessing general perceptions as well as potential demographic variations, this research contributes to a more comprehensive and culturally grounded understanding of SP practices. In doing so, it aims to broaden the global discourse on sport psychology and inform more inclusive, culturally sensitive approaches to practice and implementation.

## Materials and methods

### Participants

A total of 744 participants took part in a cross-sectional study by responding to an online survey hosted on Qualtrics (Provo, UT). Of these, 587 were fully completed, usable surveys. The final sample for this study (*n* = 587) comprised 294 athletes and 293 coaches. Participants (male = 322; 54.86%; female = 265; 45.14%) represented both individual (*n* = 241; 41.06%) and team sports (*n* = 346; 58.94%), across National/International as elite (*n* = 291; 49.6%) and Provincial/Regional as non-elite (*n* = 296; 50.4%) competitive levels. Athletes’ age ranged from 18 to 70 (*M* = 25.36, *SD* = 8.26), while coaches’ age ranged from 18 to 75 (*M* = 41.73, *SD* = 12.36). The sports most represented by the sample were: volleyball (16.7%), soccer (16%), gymnastics (11.3%), water polo (10.6%) and swimming (10.1%). Athletes and coaches were classified as elite (*Provincial, Regional*) and non-elite (*National, Internationa*l) based on Swann et al. (2015) classification. Of the participants, 35% of the athletes and 55% of the coaches had been previously exposed to SP. Additional participants’ demographics are given in Table 1.

**Table 1:** Demographic characteristics of participants.

Baseline characteristic	Athletes		Coaches	
	*n*	%	*n*	%
Gender				
Female	153	52.0	112	38.2
Male	141	48.0	181	61.8
Nationality				
Italy	291	99.0	291	99.3
Other	1	0.3	1	0.3
Did not specify	2	0.7	1	0.3
Level of competitiveness				
Provincial	36	12.2	65	22.2
Regional	94	32	101	34.5
National	145	49.3	107	36.5
International	19	6.5	20	6.8
Exposure to Sports Psych.				
Yes	102	35	162	55
No	192	65	131	45

### Research design

Prior to beginning the study, we received the ethical approval from the Institutional Review Board. Following the approval, we recruited participants by emailing administrations of different Italian sport governmental bodies (e.g., CONI, UISP), posting on multiple sport-specific social media groups (e.g., “Volley – Gruppo tecnico per gli Allentori di Pallavolo” – 13,000 subscribers), and by engaging in a snowball sampling method. To be eligible for the study, participants had to be athletes or coaches aged 18 or older, residing in Italy. If not Italian citizens, participants were asked how long they resided in Italy for. Participants who had resided in Italy for fewer than 5 years were excluded. They were required to be actively competing at the regional, national, or higher level and fluent in Italian. Upon providing informed consent, participants were invited to complete the survey.

### Instrumentation

To ensure an accurate Italian translation of the instruments, we first applied Brislin’s back-translation method (Brislin, 1970, 1986). Two independent bilingual translators employed Brislin’s back-translation method to ensure the conceptual and linguistic equivalence of the instruments translated from English to Italian. The first translator conducted the forward translation, while the second performed the back-translation. Discrepancies between the original and back-translated versions were discussed and resolved to produce a culturally and semantically accurate Italian version. The finalized translated version was used to conduct a brief pilot study with 10 random participants. Feedback from participants was used to identify and resolve unclear wording and workflow of the test. Following this process, participants completed a survey that included eight demographic questions and either the SPA-R (Martin et al., 2002) for athletes or the SPARC-2 (Zakrajsek et al., 2011) for coaches. The survey included 33 items for athletes and 34 for coaches. Both instruments are established assessments aimed at providing insight into attitudes toward sport psychology and have been utilized across different cultural contexts (Martin et al., 2004; Zakrajsek et al., 2011; Guinto-Adviento, 2016).

#### Demographical questionnaire

The initial demographic questionnaire was comprised of 8 questions. The first 4 demographic questions included, respectively, gender, age, nationality, and level of competitiveness. Gender was defined as either male, female, non-binary, and an option for those subjects who preferred to specify or who do not want to disclose. Age was treated as a continuous variable to account for the skewed distribution—characterized by a higher proportion of younger individuals and fewer older ones—ensuring that the analysis accurately captured the underlying structure of the data. A scale ranging from 18 years old up to 75+ years old was used. We limited the age scale to 75+ as a broad range due to low frequency in older age brackets. If a different nationality than Italian was reported, a question on the time residing in Italy would pop-up. The level of competitiveness followed the classification provided by Swann et al. (2015) The final 3 questions asked to indicate the sport, if participant has had previous exposures to SP, and whether participants is an athlete or coach.

#### Sport psychology attitude—revised

The sport psychology attitude—revised (SPA-R) (Martin et al., 2002) is a 25-item self-report questionnaire assessing four factors: Stigma Tolerance (ST, 7 items), Confidence in Sport Psychology (SPC, 8 items), Personal Openness (PO, 6 items), and Cultural Preference (CP, 4 items). These factors measure: (1) ST: athletes’ perception of how others associate psychological challenges with them; (2) SPC: belief in the efficacy of sport psychology and mental skills training; (3) PO: willingness to share personal concerns; and (4) CP: identification with and preference for an SP of similar cultural background. In the context of the study, the cultural homogeneity of the Italian sample may have limited variability in responses in the CP subscale. Responses are recorded on a 7-point Likert scale (1 = *strongly disagree*, 7 = *strongly agree*), with subscale scores averaged. Higher ST scores indicate a negative perception. Original reliability tests yielded Cronbach’s alpha values of 0.84 (ST), 0.82 (SPC), 0.61 (PO), and 0.66 (CP). The last two scales are considered tolerable even though their Cronbach’s coefficient is in the 0.60 range due to the number of items for each scale (Martin et al., 2002).

#### Sport psychology attitude—revised coaches

The sport psychology attitude—revised coaches (SPARC-2) is a 26-item self-report questionnaire based on the SPA-R and the Sports Psychology Attitudes—Revised Coaches (SPARC; Zakrajsek and Zizzi, 2007). It follows the SPA-R’s four-factor model, assessing (a) Stigma Tolerance (7 items), (b) Confidence in SP Consultation (8 items), (c) Personal Openness (5 items), and (d) Cultural Preference (6 items) using a 6-point Likert scale (1 = strongly disagree to 6 = strongly agree). Scoring mirrors the SPA-R, with subscale scores averaged, and higher Stigma Tolerance scores indicating a negative perception. Cronbach’s alphas demonstrated good to excellent reliability: 0.90 (Stigma Tolerance), 0.87 (Confidence in SP Consultation), 0.82 (Cultural Preference), and 0.79 (Personal Openness) (Zakrajsek and Zizzi, 2007). While the SPARC-2 has not been validated in other languages, it has been applied across various sports (Zakrajsek et al., 2011; Michel, 2013; Guinto-Adviento, 2016), competition levels (Nelson, 2008; Michel, 2013), genders (Zakrajsek and Zizzi, 2007; Zakrajsek et al., 2011), and cultural backgrounds (Guinto-Adviento, 2016). Translation of the instruments and questionnaire in Supplemental material.

### Analysis of data

We conducted a Confirmatory Factor Analysis (CFA) to evaluate the hypothesized four-factor structure of the Italian versions of the SPA-R and SPARC-2 instruments. To select the optimal model, we compared the null model (H₀) with the full model (H₁), which included all relevant predictors. The full model demonstrated a better fit, as indicated by lower values of the Akaike Information Criterion (AIC) and the Bayesian Information Criterion (BIC) (Burnham and Anderson, 2004; Kuha, 2004; Chakrabarti and Ghosh, 2011).

We then assessed model fit using multiple goodness-of-fit indices informed by distinct theoretical frameworks (Bentler, 1990; Hu and Bentler, 1999; Schreiber et al., 2006; Schumacker and Lomax, 2016; Dalila et al., 2020; Kline, 2023). Specifically, we evaluated the chi-square (χ^2^) test, the χ^2^/df ratio, the Comparative Fit Index (CFI), and the Tucker–Lewis Index (TLI). Following established guidelines, we considered CFI and TLI values ≥ 0.90, a χ^2^/df ratio between 2 and 5, and Root Mean Square Error of Approximation (RMSEA) and Standardized Root Mean Square Residual (SRMR) values < 0.08 as indicators of acceptable fit (Kline, 2023). Given the χ^2^ test’s sensitivity to large sample sizes, we emphasized RMSEA and SRMR values, with < 0.08 considered acceptable and < 0.05 indicating excellent fit (RMSEA and SRMR < 0.08 = acceptable; < 0.05 excellent).

To assess construct reliability and validity, we calculated composite reliability (CR), and average variance extracted (AVE). CR evaluated internal consistency, while AVE assessed convergent validity by quantifying the proportion of variance explained by each latent construct relative to measurement error (Dalila et al., 2020; Mohd Dzin and Lay, 2021; dos Santos and Cirillo, 2023; Cheung et al., 2024). We considered factor loadings > 0.40, AVE > 0.50, and CR > 0.70 as acceptable benchmarks (Cheung et al., 2024). We performed CFA using the *lavaan* package in R (version 4.4.2), and we computed CR and AVE values in StataIC 13.

Due to sample size limitations, we did not perform multivariate analyses across demographic variables. Instead, we applied targeted statistical methods based on questionnaire responses. We used unpaired two-tailed t-tests (*p* < 0.05) to assess gender differences, given binary male/female data in both the athlete and coach subgroups. We applied one-way ANOVA to examine differences across competitive levels, which included four categories. To evaluate age-related trends, we used one-way ANOVA following linear model fit as age was treated as a continuous variable. We first conducted demographical inferential analysis using IBM SPSS Statistics 29 and we validated for accuracy and conducted all inferential analysis using R (version 4.4.2).

## Results

### SPA-R

CFA of the SPA-R based on the original 4-factor model showed adequate fit, with CFI and Tucker Lewis Index (TLI) close to the 0.90 threshold. Additionally, RMSEA was below the 0.80 threshold value, whereas SRMR was above the threshold value of 0.80. The χ^2^/df ratio was 2.74 and fell within the values of indication of good fit.

We assessed CR and AVE for each factor: ST (CR = 0.891; AVE = 0.542); SPC (CR = 0.891; AVE = 0.533), PO (CR = 0.627; AVE = 0.262), and CP (CR = 0.823; AVE = 0.541). The PO factor appears to be more problematic in convergent validity and showed a CR (reliability assessment) value slightly lower than the indicated 0.70 limit, which reflects the low Cronbach’s alpha of the subscale (Martin et al., 2002). However, according to Fornell and Larcker (1981), convergent validity can still be considered adequate if composite reliability exceeds 0.6, even if AVE is below 0.5. Overall, while the SPA-R showed good-to-acceptable levels of goodness-of-fit in the Italian athlete sample based on the criteria assessed, the PO subscale appeared to follow similar reliability challenges as the original instrument.

### SPARC-2

The CFA conducted on the SPARC-2 *a priori* 4-factor model showed good fit of the model to the data. The parameters remained at the acceptable level of goodness of fit, with CFI reaching the 0.90 mark and TLI slightly below 0.90. RMSEA and SRMR scored below 0.80 (<0.80). χ^2^/df was 2.56 and indicated good fit of the model. We assessed CR and AVE for each factor: ST (CR = 0.919; AVE = 0.624), SPC (CR = 0.89; AVE = 0.505), PO (CR = 0.793; AVE = 0.438), and CP (CR = 0.950; AVE = 0.761) (Supplementary Figures S1, S2). Both CR and AVE across the four factors in SPARC-2 instrument had scores above the acceptable levels, with the PO scale scoring just below the 0.50 limit (Cheung et al., 2024). In the Italian coach sample, the PO scores were above the recommended acceptable guidelines, indicating the overall goodness-of-fit of the model for this sample. Table 2 offers the statistical results of the goodness of fit tests used in both instruments.

**Table 2:** Goodness of fit statistics for the instruments.

Fit statistics	SPA-R	SPARC-2
χ^2^ (df)	752.314* (275)	749.943* (293)
RMSEA	0.077*	0.073*
χ^2^/df ratio	2.74	2.56
CFI	0.851	0.898
TLI	0.837	0.887
SRMR	0.140	0.074

df, degrees of freedom; RMSEA, root mean squared error of approximation; CFI, comparative fit index; TLI, Tucker-Lewis index; SRMR, standardized root mean squared residual.

*p-value significant at the *p* < 0.001 level.

### Overall attitude

We examined the overall mean scores of each SPA-R and SPARC-2 subscale to gain a general understanding of participants’ attitudes toward sport psychology before exploring specific demographic-driven patterns. Athletes scored low on ST and on CP (M_ST_ = 2.11; M_CP_ = 2.63), high on SPC (M_SPC_ = 5.59) and moderately high on PO (M_PO_ = 3.77). Overall, it appeared that Italian athletes display low stigma, favorable attitudes toward SP services, and confidence in the effectiveness of SP (Table 3). Coaches, similarly, showed low ST and CP scores (M_ST_ = 1.66; M_CP_ = 2.68), but had high confidence in SP (M_SPC_ = 5.22). However, they scored relatively low on PO (M_PO_ = 2.94), suggesting reluctance to discuss personal issues openly (Table 3).

**Table 3:** Descriptive statistics for each subscale of the SPA-R and SPARC-2.

Subscales	SPA-R		SPARC-2	
	*M*	*SD*	*M*	*SD*
Stigma tolerance	2.11	1.08	1.66	0.90
Confidence in SP	5.59	1.00	5.22	0.75
Personal openness	3.77	0.88	2.94	1.03
Cultural preference	2.63	1.34	2.68	1.47

SPA-R used Likert scale scored from 1 to 7. SPARC-2 used a Likert scale scored from 1 to 6.

### Gender differences

**Figure 1:**
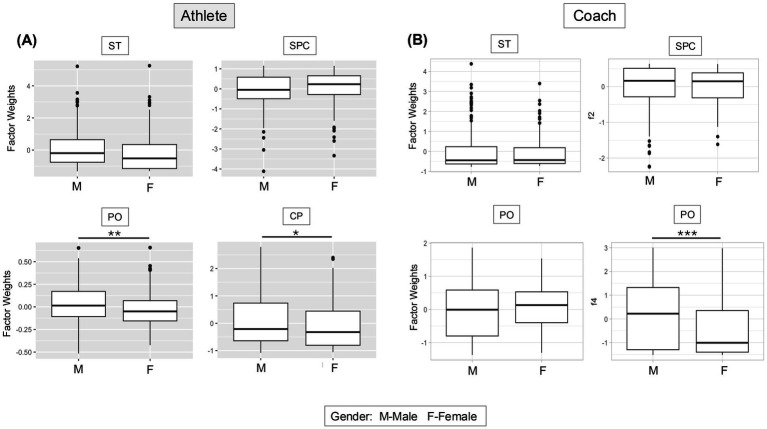
Italian female athletes and coaches show preference for SP practitioners of same cultural background. **(A,B)** Graphs displaying the unstandardized factor weights (x-axis) results for each subscale of the SPA-R **(A)** and of the SPARC-2 **(B)** by gender (y-axis). Results of the unpaired *t*-tests are shown as boxplots. * indicates *p* < 0.5, ** *p* < 0.05, *** *p* < 0.005. M = male, F = female. Further statistical analysis detail in Supplemental material. CP, Cultural Preference; PO, Personal Openness; SPC, Sport Psychology Confidence; ST, Stigma Tolerance.

Our analysis revealed significant differences between male and female athletes in the PO and CP subscales (Figure 1A). Females appear more open to discuss personal issues compared to their male counterparts (*p* = 0.008), yet less open to SP consultants of different backgrounds (*p* = 0.0417). Similarly, female coaches (Figure 1B) also prefer SP consultants of similar backgrounds (*p* < 0.001).

### Age differences

**Figure 2:**
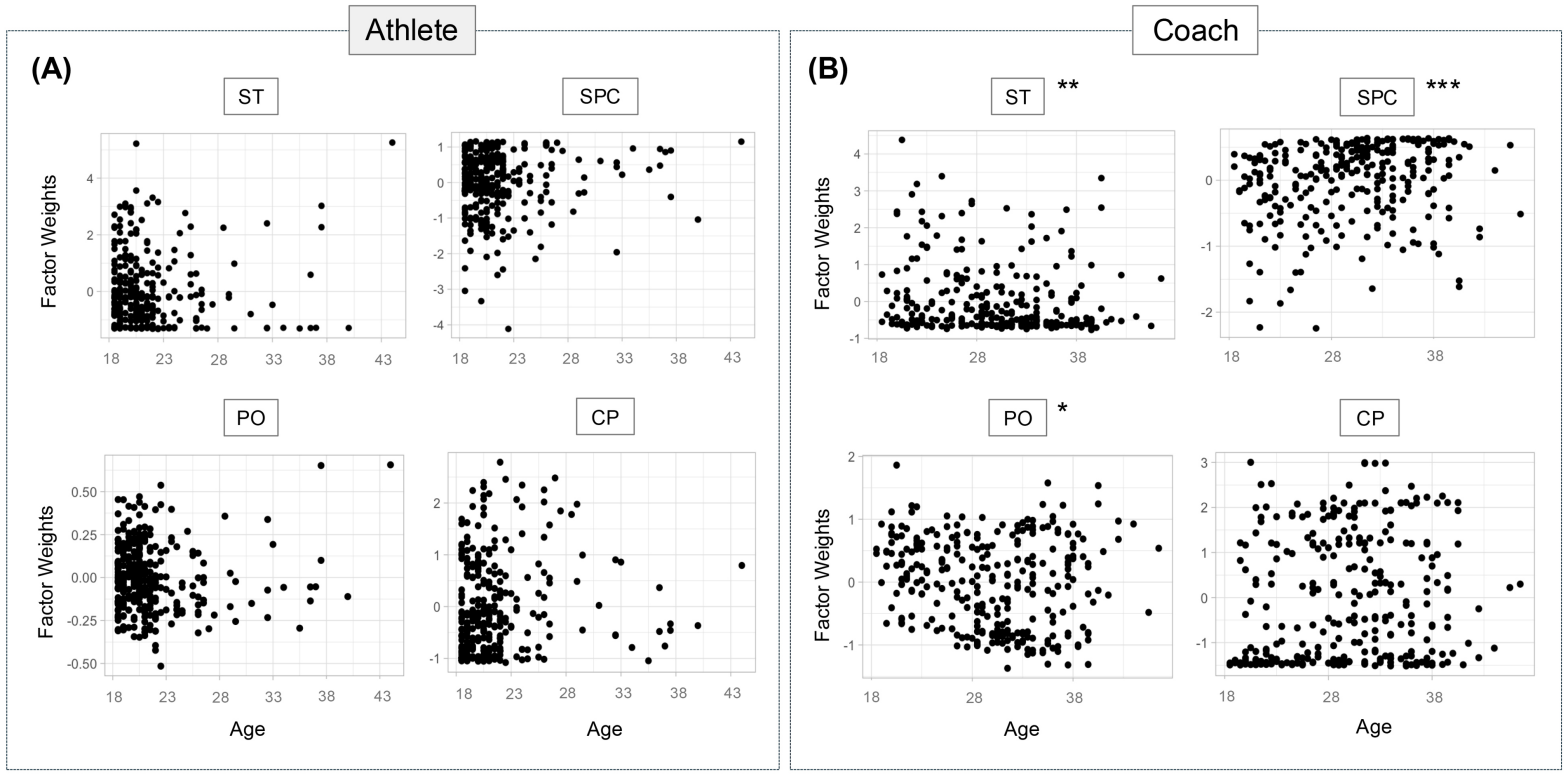
Age is a determining factor in Italian coaches’ attitude to SP. **(A,B)** Graphs displaying the unstandardized factor weights (x-axis) results for each subscale of the SPA-R **(A)** and of the SPARC-2 **(B)** by age (y-axis). Results of the linear regression analysis are shown as scatterplots. ** indicates *p* < 0.05, *** *p* < 0.005. Further statistical analysis detail in Supplemental material. CP, Cultural Preference; PO, Personal Openness; SPC, Sport Psychology Confidence; ST, Stigma Tolerance.

We identified significant age-related differences among Italian coaches, but not athletes (Figures 2A,B). For instance, ANOVA following linear model fit indicated that younger age in coaches is associated with more stigma compared to their older counterparts (*p* = 0.003). Additionally, we found that older coaches are less open to discussing personal issues compared to coaches in other age groups (*p* = 0.004). As age increases, association with confidence in sport psychology effectiveness also increases (*p* = 0.004), but we found no differences in cultural preference based on age.

### Competitive level differences

**Figure 3:**
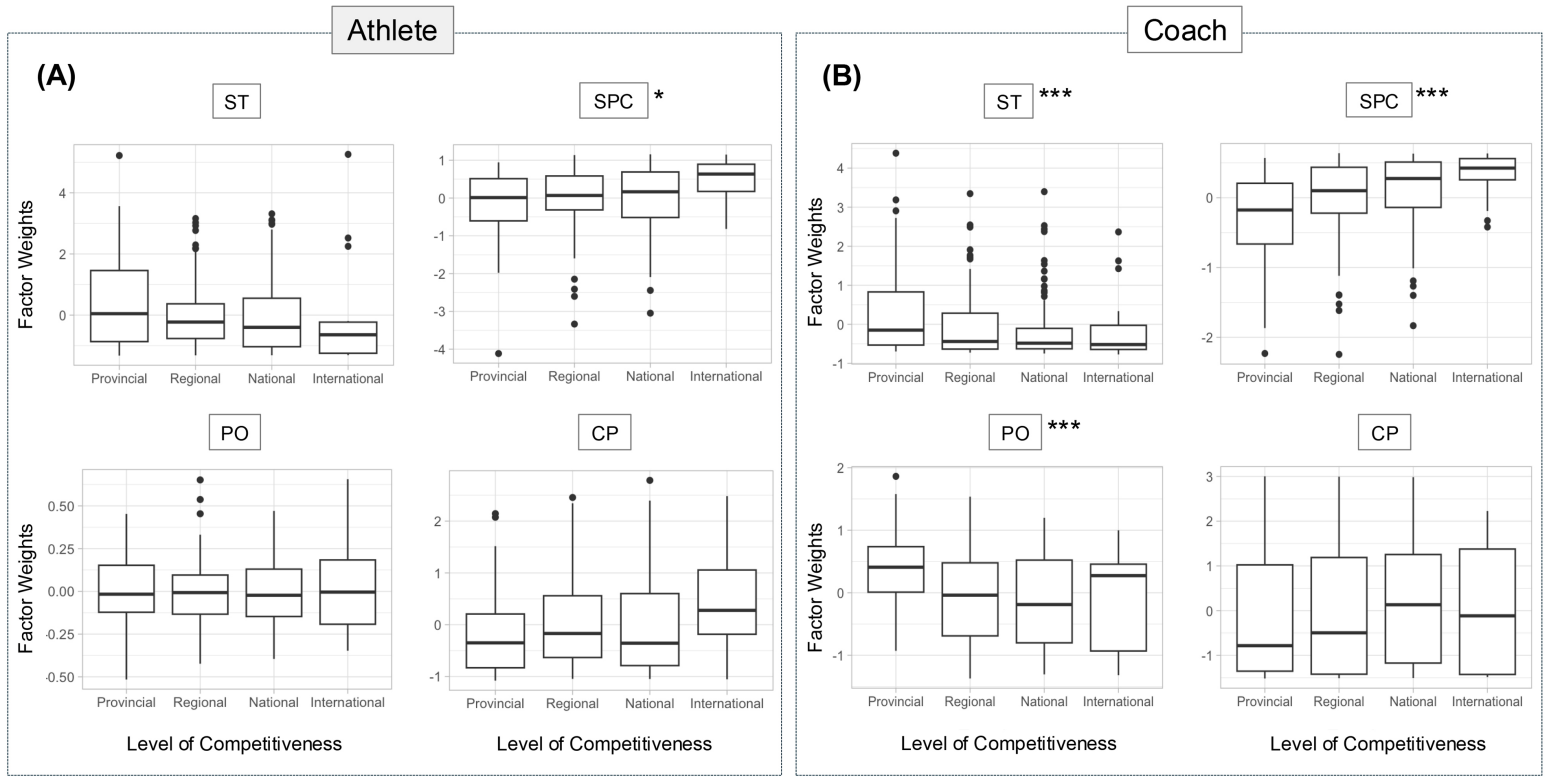
Italian non-elite coaches and athletes have less confidence in SP. **(A,B)** Graphs displaying the unstandardized factor weights (x-axis) results for each subscale of the SPA-R **(A)** and of the SPARC-2 **(B)** by level of competitiveness (y-axis). Results of the one-way ANOVA with Tuckey’s multiple comparisons are shown as boxplots. * indicates *p* < 0.5, *** *p* < 0.005. Further statistical analysis detail in Supplemental material. CP, Cultural Preference; PO, Personal Openness; SPC, Sport Psychology Confidence; ST, Stigma Tolerance.

A similar trend was seen across levels of competitiveness. Among athletes (Figure 3A), we found that confidence in the effectiveness of SP services increases as the competitive level increases (*p* = 0.031). For coaches (Figure 3B), a significant effect of the competitive level was seen for ST, SPC, and PO (*p* < 0.001). Results suggest that coaches at the provincial level possess more stigma and less confidence in the effectiveness of SP compared to their counterparts at higher competitive levels. Moreover, provincial level coaches were found to be less open to discuss personal issues compared to regional, national, and international level coaches.

## Discussion

Given the dominance of Anglo-Saxon perspectives in the SP literature, the applicability of such frameworks in other cultures remain uncertain. This study investigated how cultural factors may shape attitudes toward SP services by examining Italian athletes’ and coaches’ attitudes across gender, age, and competitive level.

CFA of the Italian version of the SPAR and SPARC supported an adequate model fit for both instruments, though the SPA-R’s Personal Openness scale showed low reliability, possibly due to response variability (Amirrudin et al., 2020). Because translation differences minimally impact psychometric properties (Detregiachi and Altran, 2023), the low reliability score of the PO subscale of the SPA-R—which fully reflects the scoring of the original subscale—might have been impacted by sample size or by variations in participant perspectives. Nonetheless, the identified low internal reliability in the PO subscale potentially limits the validity of demographic comparisons and interpretation of related findings.

Despite the PO subscale’s limitation, Italian athletes’ attitudes align with international trends, offering a foundation for cross-cultural comparisons. For example, Italian athletes reported low stigma and moderately high confidence in SP, mirroring findings from New Zealand and Spain, though with slightly lower confidence in service effectiveness (Anderson et al., 2004; Checa et al., 2023). This discrepancy may be attributed to limited access to SP services in Italy, as reflected by the fact that only 35% of athletes in the current sample had previous exposure to SP. While elite athletes may benefit from access to multidisciplinary support teams (Reid et al., 2004), there is limited evidence on the accessibility of SP services for athletes at all levels, and non-elite athletes and coaches often navigate these challenges independently (Moesch et al., 2018).

A possible limiting role in accessibility could be brought by organizational deficiencies in Italy. In Anglo-Saxon contexts, research has shown that structural issues within sports organizations hinder the delivery of SP interventions (McDougall et al., 2015). Yet, recent efforts in the UK have aimed to embed psychologically informed practices within organizational frameworks, enhancing both performance and culture (Wagstaff and Quartiroli, 2023). Drawing from the UK experience, it becomes evident that a structured and culturally informed organizational approach is essential for the effective integration of SP. Without clear frameworks or culturally sensitive practices embedded within sports organizations, the adoption and effectiveness of SP in Italy may remain limited, particularly outside elite-level contexts. Additionally, the comparatively lower confidence in SP among Italian non-elite coaches, when contrasted with their elite-level counterparts, may reflect systemic limitations in accessibility as seen in other countries (Gould et al., 1991; Gentner et al., 2004; Barker and Winter, 2014). Addressing these challenges through clearer institutional frameworks and increased organizational support is essential for fostering equitable and widespread access to SP services across all levels of Italian sport.

While Italian athletes expressed less confidence in SP effectiveness, they were more open to consulting SP practitioners from diverse cultural backgrounds than their British, American, and German counterparts (Martin et al., 2004). Over the past two decades, Italy’s foreign-born population has increased by more than 6% (ISTAT - Istituto Nazionale di Statistica, 2023), a demographic shift reflected in the sports domain, where second-generation immigrants are more actively engaged than first-generation athletes (van Tubergen and Molteni, 2024). This growing presence of foreign-born individuals—both in society and in sports—may contribute to greater openness among Italian athletes toward culturally diverse practitioners. As second-generation immigrants bring varied perspectives and lived experiences, they may foster inclusive team dynamics and promote cross-cultural exchange, with sport serving as a platform for cultural integration (Hatzigeorgiadis et al., 2013). Such multicultural exposure within training environments likely enhances athletes’ receptiveness to consulting professionals from different cultural backgrounds. This trend underscores the value of cultivating culturally diverse and inclusive support systems within sport.

However, gender-based differences emerged: Italian female athletes and coaches showed a stronger preference for consultants who shared similar cultural backgrounds. This contrasts with existing research suggesting that women in sport generally express more favorable attitudes toward SP than men (Brooks and Bull, 1999; Martin, 2005; Zakrajsek and Zizzi, 2007; Wrisberg et al., 2009, 2010). One possible explanation is a relative lack of exposure to multiculturalism within women’s sports contexts. A study by Naoi et al. (2011) suggest that cultural homogeneity may reinforce preferences for practitioners with shared identities. Moreover, research highlights the scarcity of policies and initiatives aimed at promoting cultural diversity and multicultural awareness in women’s sports (Cortis, 2009). As a result, women’s sports in Italy may be missing out on the benefits of increasing demographic diversity. The underrepresentation of women in decision-making roles—such as their minimal presence in football leadership, which barely exceeds 15% (Trequattrini et al., 2022)—may further limit the implementation of inclusive policies. These findings suggest that without intentional efforts to promote multicultural awareness, the effectiveness and reach of SP services may remain constrained, particularly for female athletes and coaches.

In contrast with the more favorable views of athletes, younger Italian coaches reported greater stigma and lower confidence in SP, consistent with findings among American coaches under 30 (Zakrajsek et al., 2011; Michel, 2013). Similarly, older Italian coaches exhibited less openness to discussing personal issues—an attitude not mirrored in Anglo-Saxon populations (Sullivan and Hodge, 1991; Byrne and Cusack, 2015). While previous literature suggests that exposure to SP can positively influence perceptions (Bell et al., 2022), the fact that over half of respondents in the current study had prior exposure, yet retained negative attitudes, indicates that cultural factors may override experiential familiarity. The Italian coaches’ attitude to SP mirrors trends observed in Eastern cultural contexts, where openness is not a strong predictor of SP receptivity due to underlying concerns about family honor and emotional restraint (Guinto-Adviento, 2016; Ong and Harwood, 2018). Similarly, Italy’s culture of honor—rooted in traditions that discourage emotional expressiveness (Vitiello, 2023)—may serve as a barrier to SP acceptance. These findings highlight the importance cultural context in shaping SP engagement (Yasuda, 2022) and identification of the root causes of this reluctance is critical, as coaches play a key role in shaping athletes’ intentions (Wekesser et al., 2021). Framed within the Theory of Planned Behavior (Bosnjak et al., 2020), these results suggest that coaches may serve as key agents in influencing subjective norms and perceived behavioral control, both of which are critical determinants of athletes’ intentions to seek SP support.

Tailoring interventions to align with cultural values and norms, particularly among coaches, may enhance acceptance and reduce stigma (Bissett et al., 2020). At the same time, younger coaches—who may hold doubts despite being more receptive—may benefit from educational content integrated into certification programs (Li et al., 2024). Such programs should emphasize the concrete benefits of SP for performance, resilience, and well-being, thereby fostering a culture of openness and trust.

Overall, these findings underscore the importance of understanding the cultural context in which SP operates. Moving beyond the predominantly Anglo-Saxon perspective, culturally informed research is essential to develop interventions that resonate with the values, norms, and expectations of diverse populations. Tailoring SP practices to specific cultural settings can enhance their relevance, effectiveness, and acceptance across multiple stakeholders. Embracing this cultural lens is essential for advancing more equitable and globally relevant SP services.

## Limitations and future research

This study has several limitations that should be acknowledged. First, the restricted age range of participants (most participants falling within an 18 to 26 years old range) limits the generalizability of our findings across different life stages and may obscure potential age-related differences in perceptions of SP. Future studies should aim to include a more diverse age spectrum. Second, the cross-sectional design paired with non-probabilistic sampling, such as online recruitment and snowball sampling, may have introduced self-selection bias, likely overrepresenting individuals with a preexisting interest in SP, and prevented the establishment of causal relationships or changes over time. Longitudinal research or mixed-methods approaches are necessary to track how attitudes toward SP evolve over time, particularly in response to targeted interventions or broader cultural shifts.

Additionally, employing more rigorous sampling strategies, such as stratified random sampling, would enhance the representativeness of future samples. Of note, while our sample size supported multiple univariate analyses, future studies employing multivariate approaches (e.g., MANOVA) could offer greater control over covariates and potential mediators.

Third, issues with the reliability of the Personal Openness subscale of the SPA-R suggest possible challenges in translation or cultural interpretation. Specifically, the SPA-R PO subscale showed low internal reliability, limiting the validity and interpretability of related findings, despite acceptable reliability across other subscales. This underscores the need for further cultural validation and potential adaptation of SP instruments in non-Anglophone contexts.

Finally, the study’s exclusive use of quantitative methodology constrained its ability to detect culturally nuanced perspective and culturally embedded aspects of SP perceptions. Incorporating qualitative methodologies in future research, such as interviews or focus groups, may facilitate a more comprehensive and contextually grounded understanding of SP attitudes.

## Conclusion

In conclusion, this study offers a culturally grounded perspective on SP by examining the perceptions of Italian athletes and coaches—a context often underrepresented in the existing literature. By addressing the prevalence of Anglo-Saxon models in SP discourse, our findings broaden the understanding of how SP is perceived across different cultures. The observed openness among Italian stakeholders underscores a promising trajectory for the integration of SP in diverse settings. These insights not only inform future SP practice in Italy but also emphasize the importance of culturally nuanced research to enhance global dialog and collaboration among sport psychology professionals.

## Data Availability

The original contributions presented in the study are included in the article/Supplementary material, further inquiries can be directed to the corresponding author.

## Supplementary material

Supplementary Figure S1 Standardized results of SPA-R showing factor loading on Italian athlete sample. Ɛ = measurement error. Covariance between Ɛ1 and Ɛ3 indicates modification index. ST, Stigma Tolerance; SPC, Confidence in Sport Psychology; PO, Personal Openness; CP, Cultural Preference.

Supplementary Figure S2 Standardized results of SPARC2 showing factor loading on Italian coach sample. Ɛ = measurement error. ST, Stigma Tolerance; SPC, Confidence in Sport Psychology; PO, Personal Openness; CP, Cultural Preference.

Supplementary Table S1 Raw data files.

Supplementary File 1 Original questionnaire.

Supplementary File 2 SPSS outputs.

Supplementary File 3 SPSS outputs.

Supplementary Data Sheet 1 Full R-analysis statistical output.
