# Supplementary figures and images for: Cultural attitudes toward sport psychology: insights from Italian athletes and coaches
Source: Front Psychol. 2025 Aug 4;16:1630005. doi: 10.3389/fpsyg.2025.1630005 (PMC12358425; doi:10.3389/fpsyg.2025.1630005)

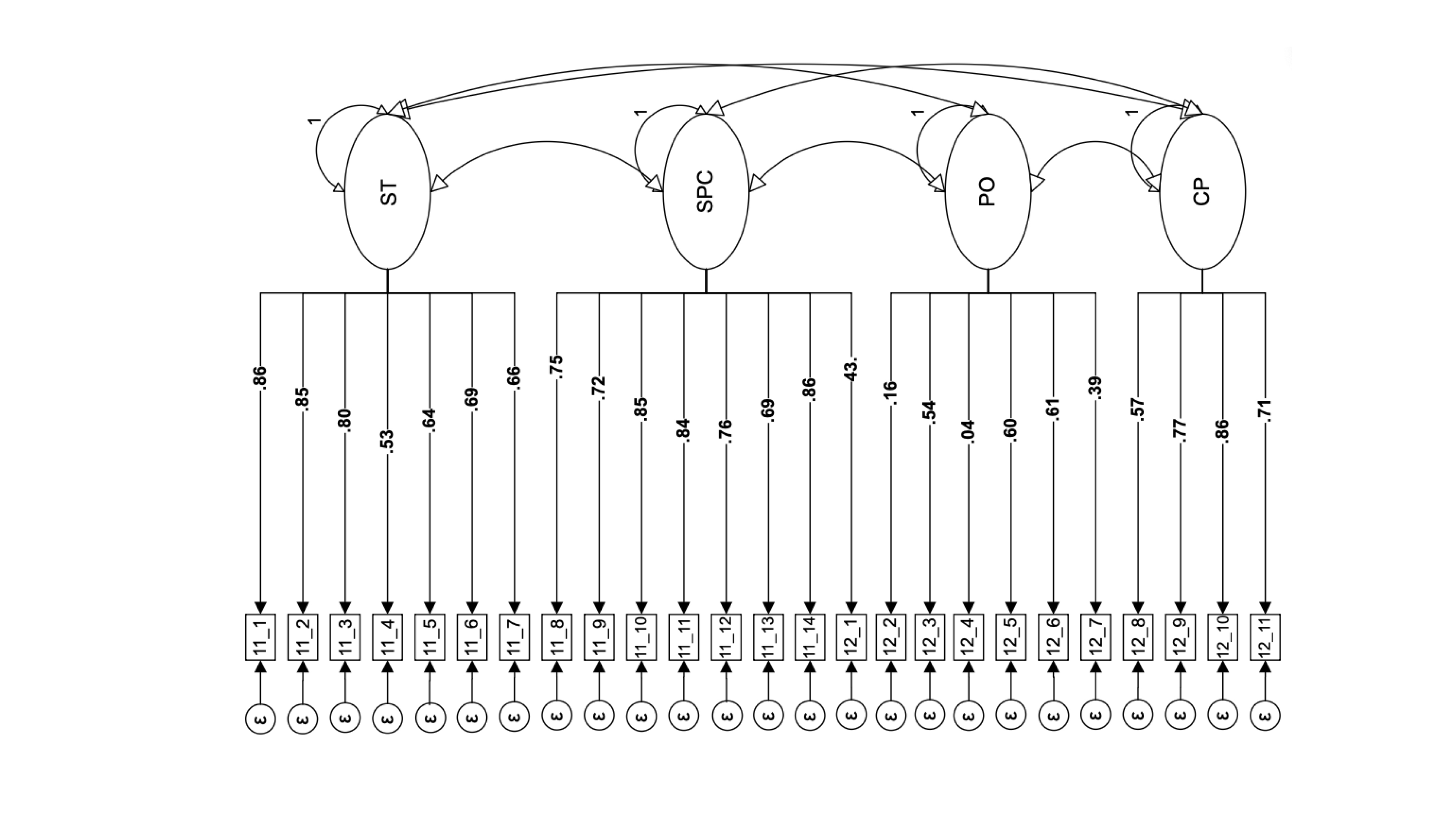

Supplement: Supplementary Figure S1 — Standardized results of SPA-R showing factor loading on Italian athlete sample. Ɛ = measurement error. Covariance between Ɛ1 and Ɛ3 indicates modification index. ST, Stigma Tolerance; SPC, Confidence in Sport Psychology; PO, Personal Openness; CP, Cultural Preference. [file Image_1.jpeg]

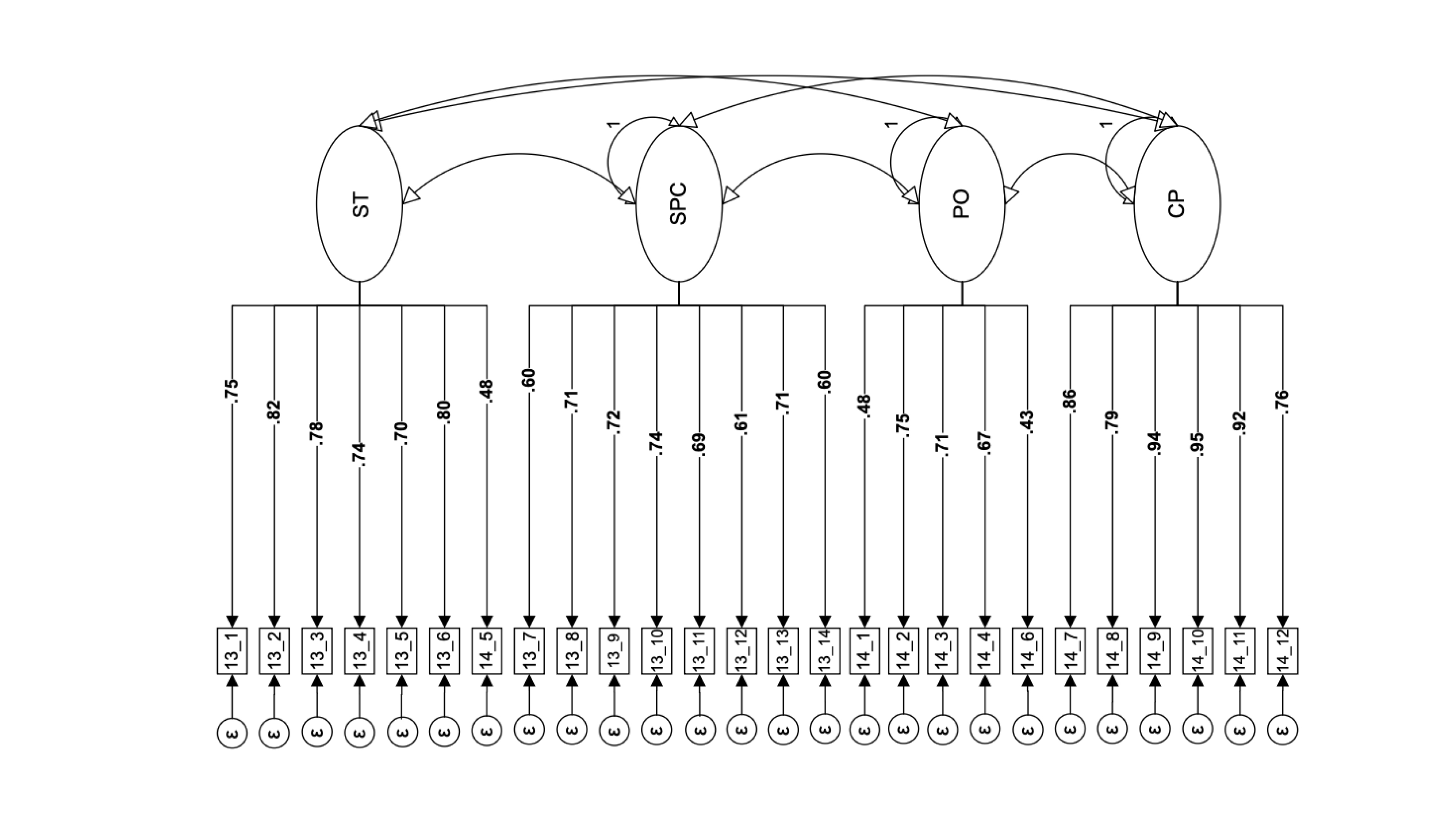

Supplement: Supplementary Figure S2 — Standardized results of SPARC2 showing factor loading on Italian coach sample. Ɛ = measurement error. ST, Stigma Tolerance; SPC, Confidence in Sport Psychology; PO, Personal Openness; CP, Cultural Preference. [file Image_2.jpeg]
