# Supplementary material for: Cultural attitudes toward sport psychology: insights from Italian athletes and coaches
Source: Front Psychol. 2025 Aug 4;16:1630005. doi: 10.3389/fpsyg.2025.1630005 (PMC12358425; doi:10.3389/fpsyg.2025.1630005)
Supplement: Supplementary File 1 — Original questionnaire. [file Supplementary_file_1.docx]

**Table 1**

*Demographical questionnaire.*

| **Gender** | Maschio | Femmina | Non-Binario | Preferisco specificarlo: | Preferisco non divulgarlo |
| --- | --- | --- | --- | --- | --- |
| **Età** |  |  |  |  |  |
| **Nazionalità** |  |  |  |  |  |
| **Tempo passato in Italia (*se non Nazionalità: *Italia*)** |  |  |  |  |  |
| **Livello di competizione** | Regionale | Provinciale | Nazionale | Internazionale |  |

| **Sport** |  |
| --- | --- |
| ***Individuale*** | ***Team*** |
| Tennis | Calcio/Futsal |
| Ginnastica | Pallavolo |
| Danza | Basket |
| Sci | Pallanuoto |
| Nuoto | Rugby |
| Corsa/Atletica | Pallamano |
| Ciclismo | Hockey |
| Golf | Canottaggio |
| Pattinaggio (ghiaccio e non) | Baseball |
| Boxe | Curling |
| Tuffi | Altro: |

| **Sei mai stat* espost* alla psicologia sportiva?** | Si | No |
| --- | --- | --- |
| **Sei un* atleta o un* coach?** | Atleta | Coach |

**Table 1**

*English-Translated Demographical questionnaire.*

| **Gender** | Male | Female | Non-Binary | Prefer to specify: | Prefer not to say |
| --- | --- | --- | --- | --- | --- |
| **Age** |  |  |  |  |  |
| **Nationality** |  |  |  |  |  |
| **Time spent in Italy (if *Nationality* not Italian)** |  |  |  |  |  |
| **Level of Competitveness** | Regional | Provincial | National | International |  |

| **Sport** |  |
| --- | --- |
| ***Individual*** | ***Team*** |
| Tennis | Soccer/Futsal |
| Gymnastics | Volleyball |
| Dance | Basketball |
| Ski | Water Polo |
| Swimming | Rugby |
| Track and Field | Handball |
| Biking | Hockey |
| Golf | Rowing |
| Skating (Ice and non-ice skating) | Baseball |
| Boxe | Curling |
| Diving | Other: |

| **Have you been previously exposed to SP?** | Yes | No |
| --- | --- | --- |
| **Are you an athlete or a coach?** | Athlete | Coach |

**Table 2**

*SPA-R items.* 
Indica cortesemente la tua opinione sulle seguenti affermazioni cliccando la risposta che meglio corrisponde al tuo pensiero riguardo ogni asserzione. Non ci sono risposte giuste o sbagliate. Usando la scala seguente, ti chiediamo di rispondere alle asserzioni nel modo più veritiero possibile. 
 
NOTA: PS = psicologo/a dello sport

| **1- FD      Fortemente in disaccordo** | **2-D**  **In disaccordo** | **3-MD Moderatamente d’accordo** | **4-N Neutrale** | **5-MA Moderatamente in accordo** | **6-A      In accordo** | **7-FA Fortemente in accordo** |
| --- | --- | --- | --- | --- | --- | --- |

| **Stigma Tolerance** |  |  |  |  |  |  |  |
| --- | --- | --- | --- | --- | --- | --- | --- |
|  |  |  |  |  |  |  |  |
| **04.** Non voglio che qualcuno sappia che ricevo aiuto da un* PS. | 1 | 2 | 3 | 4 | 5 | 6 | 7 |
| **09.** Non voglio che il/la mio/a allenatore/allenatrice sappia che sono andat* a un* PSs. | 1 | 2 | 3 | 4 | 5 | 6 | 7 |
| **13.** Non voglio che altri/e atleti/e sappiano che sono andat* da un* PS. | 1 | 2 | 3 | 4 | 5 | 6 | 7 |
| **18.** L'allenatore/allenatrice mi rispetterebbe meno se andassi da un* PS. | 1 | 2 | 3 | 4 | 5 | 6 | 7 |
| **19.** Vedere un* PS è dannoso per la reputazione di un* atlet*. | 1 | 2 | 3 | 4 | 5 | 6 | 7 |
| **21.** Non andrei da un* PS perché un altro/a atleta mi assillerebbe. | 1 | 2 | 3 | 4 | 5 | 6 | 7 |
| **23.** Non andrei da un* PS perché un altr* atleta mi assillerebbe. | 1 | 2 | 3 | 4 | 5 | 6 | 7 |
|  |  |  |  |  |  |  |  |
| **Confidence in SPC** |  |  |  |  |  |  |  |
|  |  |  |  |  |  |  |  |
| **01.**Un* PS potrebbe aiutare a competere meglio sotto pressione. | 1 | 2 | 3 | 4 | 5 | 6 | 7 |
| **03.** Cercherei il supporto di un* PS se fossi deluso dalle mie prestazioni. | 1 | 2 | 3 | 4 | 5 | 6 | 7 |
| **08.** Apprezzerei il supporto di un* PS per capire meglio me stesso. | 1 | 2 | 3 | 4 | 5 | 6 | 7 |
| **12**. Un* PS potrebbe aiutarmi a perfezionare le mie prestazioni sportive. | 1 | 2 | 3 | 4 | 5 | 6 | 7 |
| **15.** Un* PS può aiutare a migliorare la forza mentale. | 1 | 2 | 3 | 4 | 5 | 6 | 7 |
| **17.** Mi sono sentit* pers* e avrei ricevuto volentieri del supporto professionale. | 1 | 2 | 3 | 4 | 5 | 6 | 7 |
| **20.** Mi sentirei più sicur* se ricevessi il supporto di un* PS | 1 | 2 | 3 | 4 | 5 | 6 | 7 |
| **22.** Se un* compagn* di squadra me lo chiedesse, potrei indicare un* PS. | 1 | 2 | 3 | 4 | 5 | 6 | 7 |
|  |  |  |  |  |  |  |  |
| **Personal Openness** |  |  |  |  |  |  |  |
|  |  |  |  |  |  |  |  |
| **05.** Un buon modo di evitare preoccupazioni è di mantenere la testa sul lavoro. | 1 | 2 | 3 | 4 | 5 | 6 | 7 |
| **07.** Atlet* con un carattere forte superano da sol* le proprie difficoltà. | 1 | 2 | 3 | 4 | 5 | 6 | 7 |
| **10.** È rispettabile la volontà di far fronte alle proprie difficoltà. | 1 | 2 | 3 | 4 | 5 | 6 | 7 |
| **14.** Le difficoltà emotive tendono a risolversi da sole nel tempo. | 1 | 2 | 3 | 4 | 5 | 6 | 7 |
| **16.** Certi problemi non dovrebbero essere discussi al di fuori della propria famiglia. | 1 | 2 | 3 | 4 | 5 | 6 | 7 |
| **24.** Ci sono esperienze nella mia vita di cui non parlerei con nessuno. | 1 | 2 | 3 | 4 | 5 | 6 | 7 |
|  |  |  |  |  |  |  |  |
| **Cultural Preference** |  |  |  |  |  |  |  |
|  |  |  |  |  |  |  |  |
| **02.** Rispetto le opinioni/costumi della cultura a cui appartengo più di quelle di altre culture. | 1 | 2 | 3 | 4 | 5 | 6 | 7 |
| **06.** Ci sono grandi differenze tra persone di razze diverse. | 1 | 2 | 3 | 4 | 5 | 6 | 7 |
| **11.** MI sentirei più a mio agio con un* PS se fosse della mia stessa etnia. | 1 | 2 | 3 | 4 | 5 | 6 | 7 |
| **25.** Gli/Le atleti/e con cui mi associo sono della mia stessa razza. | 1 | 2 | 3 | 4 | 5 | 6 | 7 |

*Note:* Bold numbers before survey items indicate the rank in which the items were presented in original survey.

**Table 3.**

*SPARC-2 items.* 
Indica cortesemente la tua opinione sulle seguenti affermazioni cliccando la risposta che meglio corrisponde al tuo pensiero riguardo ogni asserzione. Non ci sono risposte giuste o sbagliate. Usando la scala seguente, ti chiediamo di rispondere alle asserzioni nel modo più veritiero possibile. 
 
NOTA: PS = psicologo/a dello sport

| 1. **FD**   **Fortemente in disaccordo** | **2-D**  **In disaccordo** | **3-MD Moderatamente d’accordo** | **4-N Neutrale** | **5-MA Moderatamente in accordo** | **6-A**  **In accordo** | **7-FA Fortemente in accordo** |
| --- | --- | --- | --- | --- | --- | --- |

| **Stigma Tolerance** |  |  |  |  |  |  |
| --- | --- | --- | --- | --- | --- | --- |
|  |  |  |  |  |  |  |
| **03.** Non vorrei che un* PS lavorasse con le/i mie/i atlete/i perché mi screditerebbe agli occhi di altri/e allenatori/allenatrici | 1 | 2 | 3 | 4 | 5 | 6 |
| **06.** Mi sentirei a disagio ad avere un* PS che lavori con le/i mie/i atlete/i perché alcune persone potrebbero non approvare. | 1 | 2 | 3 | 4 | 5 | 6 |
| **08.** Se usassi un* PS per aiutarmi ad allenare meglio, non vorrei che gli/le altri/e allenatori/allenatrici lo sapessero. | 1 | 2 | 3 | 4 | 5 | 6 |
| **15.** Non vorrei che si sapesse che le/i mie/i atlete/i ricevono il supporto di un* PS. | 1 | 2 | 3 | 4 | 5 | 6 |
| **10.** Aver consultato un* PS danneggia la reputazione di un* atleta. | 1 | 2 | 3 | 4 | 5 | 6 |
| **18.** Se le/i mie/i atlete/i lavorassero con un* PS, non vorrei che altri allenatori/allenatrici lo sapessero. | 1 | 2 | 3 | 4 | 5 | 6 |
| **21.** Rispetterei meno delle/dei mie/i atlete/i se consultassero un* PS | 1 | 2 | 3 | 4 | 5 | 6 |
|  |  |  |  |  |  |  |
| **Confidence in SPC** |  |  |  |  |  |  |
|  |  |  |  |  |  |  |
| **14.** Penso che un* PS aiuterebbe le/i mie/i atlete/i a competere meglio sotto pressione. | 1 | 2 | 3 | 4 | 5 | 6 |
| **02.** Se un* atleta nella mia squadra mi chiedesse un consiglio rispetto al proprio senso di fallimento relativo allo sport, io suggerirei di consultare un* PS. | 1 | 2 | 3 | 4 | 5 | 6 |
| **05.** Apprezzerei l'assistenza di un* PS per aiutarmi a capire meglio le/i mie/i atlete/i. | 1 | 2 | 3 | 4 | 5 | 6 |
| **09.** Ritengo che un* atleta con problemi emotivi durante la prestazione sportiva si sentirebbe più sicuro nel ricevere assistenza da un* SP. | 1 | 2 | 3 | 4 |  | 6 |
| **01.**Un* PS può aiutare le/gli atlete/i a migliorare la propria forza mentale. | 1 | 2 | 3 | 4 | 5 | 6 |
| **11.** Se fossi preoccupato o deluso per le prestazioni delle/dei mie/i atlete/i, chiedere il supporto di un* PS. | 1 | 2 | 3 | 4 | 5 | 6 |
| **17.** Un* PS potrebbe aiutare le/i mie/i atlete/i a perfezionare le loro prestazioni. | 1 | 2 | 3 | 4 | 5 | 6 |
| **20.** A volte mi sono sentito perso e avrei accolto un consiglio professionale per un problema personale. | 1 | 2 | 3 | 4 | 5 | 6 |
|  |  |  |  |  |  |  |
| **Personal Openness** |  |  |  |  |  |  |
|  |  |  |  |  |  |  |
| **04.** Una buona soluzione per un* atleta per evitare preoccupazioni e problemi personali è di concentrarsi sul proprio lavoro | 1 | 2 | 3 | 4 | 5 | 6 |
| **07.** C'è qualcosa di rispettabile nell'atteggiamento di atleti/e che sono disposti ad affrontare i loro conflitti e paure senza ricorrere ad un supporto professionale. | 1 | 2 | 3 | 4 | 5 | 6 |
| **13.** Le difficoltà emotive degli/delle atleti/e tendono a risolversi nel tempo. | 1 | 2 | 3 | 4 | 5 | 6 |
| **22.** Atleti/e forti di carattere possono superare da sol* i propri conflitti mentali | 1 | 2 | 3 | 4 | 5 | 6 |
| **25.** Atleti/e dovrebbero sapere come gestire i problemi senza aver bisogno dell'assistenza di un* PS. | 1 | 2 | 3 | 4 | 5 | 6 |
|  |  |  |  |  |  |  |
| **Cultural Preference** |  |  |  |  |  |  |
|  |  |  |  |  |  |  |
| **12.** Se dovessi assumere un* PS, prenderei in considerazione la sua razza o etnia. | 1 | 2 | 3 | 4 | 5 | 6 |
| **16.** Ci sono grandi differenze tra persone di diverse razze o etnie. | 1 | 2 | 3 | 4 | 5 | 6 |
| **19.** Le/I mie/i atlete/i sarebbero più a loro agio con un* PS se fosse della loro stessa razza o etnia. | 1 | 2 | 3 | 4 | 5 | 6 |
| **23.** Mi sentirei più a mio agio con uno/a PS se fosse della mia stessa razza o etnia. | 1 | 2 | 3 | 4 | 5 | 6 |
| **24.** Un/a atleta può relazionarsi meglio con un* PS se fosse della sua stessa razza o etnia. | 1 | 2 | 3 | 4 | 5 | 6 |
| **26.** Mi sentirei più a mio agio nell'assumere un* PS se provenisse dallo stesso contesto culturale delle/i mie/i atlete/i. | 1 | 2 | 3 | 4 | 5 | 6 |

*Note:* Bold numbers before survey items indicate the rank in which the items were presented in original survey.
