# Supplementary material for: Cultural attitudes toward sport psychology: insights from Italian athletes and coaches
Source: Front Psychol. 2025 Aug 4;16:1630005. doi: 10.3389/fpsyg.2025.1630005 (PMC12358425; doi:10.3389/fpsyg.2025.1630005)
Supplement: Supplementary File 2 — SPSS outputs. [file Supplementary_file_2.docx]

**Frequencies**

| **Notes** |  |  |
| --- | --- | --- |
| Output Created |  | 20-DEC-2024 11:42:23 |
| Comments |  |  |
| Input | Data | \\apporto.com\dfs\GWU\Users\pankratimenos_gwu\Desktop\SPSSData.sav |
|  | Active Dataset | DataSet2 |
|  | Filter | <none> |
|  | Weight | <none> |
|  | Split File | <none> |
|  | N of Rows in Working Data File | 594 |
| Missing Value Handling | Definition of Missing | User-defined missing values are treated as missing. |
|  | Cases Used | Statistics are based on all cases with valid data. |
| Syntax |  | FREQUENCIES VARIABLES=ST /NTILES=4 /STATISTICS=STDDEV VARIANCE MINIMUM MAXIMUM MEAN MEDIAN /HISTOGRAM /ORDER=ANALYSIS. |
| Resources | Processor Time | 00:00:00.17 |
|  | Elapsed Time | 00:00:00.13 |

| **Statistics** |  |  |
| --- | --- | --- |
| ST |  |  |
| N | Valid | 297 |
|  | Missing | 297 |
| Mean |  | 2.1075 |
| Median |  | 1.8571 |
| Std. Deviation |  | 1.08307 |
| Variance |  | 1.173 |
| Minimum |  | 1.00 |
| Maximum |  | 7.00 |
| Percentiles | 25 | 1.2857 |
|  | 50 | 1.8571 |
|  | 75 | 2.5714 |

| **ST** |  |  |  |
| --- | --- | --- | --- |
|  |  | N | % |
| 1.00 |  | 54 | 9.1% |
| 1.14 |  | 13 | 2.2% |
| 1.29 |  | 13 | 2.2% |
| 1.43 |  | 26 | 4.4% |
| 1.57 |  | 16 | 2.7% |
| 1.71 |  | 16 | 2.7% |
| 1.86 |  | 14 | 2.4% |
| 2.00 |  | 30 | 5.1% |
| 2.14 |  | 13 | 2.2% |
| 2.20 |  | 1 | 0.2% |
| 2.29 |  | 13 | 2.2% |
| 2.43 |  | 9 | 1.5% |
| 2.57 |  | 7 | 1.2% |
| 2.71 |  | 12 | 2.0% |
| 2.86 |  | 5 | 0.8% |
| 3.00 |  | 6 | 1.0% |
| 3.14 |  | 5 | 0.8% |
| 3.29 |  | 2 | 0.3% |
| 3.43 |  | 4 | 0.7% |
| 3.57 |  | 6 | 1.0% |
| 3.71 |  | 2 | 0.3% |
| 3.86 |  | 7 | 1.2% |
| 4.00 |  | 3 | 0.5% |
| 4.14 |  | 2 | 0.3% |
| 4.29 |  | 6 | 1.0% |
| 4.43 |  | 1 | 0.2% |
| 4.57 |  | 3 | 0.5% |
| 4.71 |  | 1 | 0.2% |
| 4.86 |  | 2 | 0.3% |
| 5.00 |  | 1 | 0.2% |
| 5.29 |  | 1 | 0.2% |
| 5.43 |  | 1 | 0.2% |
| 7.00 |  | 2 | 0.3% |
| Missing | System | 297 | 50.0% |
|  |  |  |  |
|  |  |  |  |

**Frequencies**

| **Notes** |  |  |
| --- | --- | --- |
| Output Created |  | 20-DEC-2024 11:46:26 |
| Comments |  |  |
| Input | Data | \\apporto.com\dfs\GWU\Users\pankratimenos_gwu\Desktop\SPSSData.sav |
|  | Active Dataset | DataSet2 |
|  | Filter | <none> |
|  | Weight | <none> |
|  | Split File | <none> |
|  | N of Rows in Working Data File | 594 |
| Missing Value Handling | Definition of Missing | User-defined missing values are treated as missing. |
|  | Cases Used | Statistics are based on all cases with valid data. |
| Syntax |  | FREQUENCIES VARIABLES=SPC PO CP /NTILES=4 /STATISTICS=STDDEV VARIANCE MINIMUM MAXIMUM MEAN MEDIAN /HISTOGRAM /ORDER=ANALYSIS. |
| Resources | Processor Time | 00:00:00.69 |
|  | Elapsed Time | 00:00:00.33 |

| **Statistics** |  |  |  |  |
| --- | --- | --- | --- | --- |
|  |  | SPC | PO | CP |
| N | Valid | 298 | 297 | 297 |
|  | Missing | 296 | 297 | 297 |
| Mean |  | 5.5853 | 3.7668 | 2.6296 |
| Median |  | 5.7500 | 3.8333 | 2.2500 |
| Std. Deviation |  | 1.00151 | .87226 | 1.33686 |
| Variance |  | 1.003 | .761 | 1.787 |
| Minimum |  | 1.00 | 1.00 | 1.00 |
| Maximum |  | 7.00 | 6.00 | 6.75 |
| Percentiles | 25 | 5.0000 | 3.1667 | 1.5000 |
|  | 50 | 5.7500 | 3.8333 | 2.2500 |
|  | 75 | 6.3750 | 4.3333 | 3.5000 |

**Frequency Table**

| **SPC** |  |  |  |
| --- | --- | --- | --- |
|  |  | N | % |
| 1.00 |  | 1 | 0.2% |
| 2.00 |  | 1 | 0.2% |
| 2.13 |  | 1 | 0.2% |
| 2.63 |  | 2 | 0.3% |
| 3.00 |  | 2 | 0.3% |
| 3.13 |  | 1 | 0.2% |
| 3.25 |  | 1 | 0.2% |
| 3.38 |  | 3 | 0.5% |
| 3.75 |  | 3 | 0.5% |
| 3.88 |  | 8 | 1.3% |
| 4.00 |  | 4 | 0.7% |
| 4.13 |  | 1 | 0.2% |
| 4.25 |  | 4 | 0.7% |
| 4.38 |  | 5 | 0.8% |
| 4.50 |  | 4 | 0.7% |
| 4.63 |  | 6 | 1.0% |
| 4.75 |  | 10 | 1.7% |
| 4.88 |  | 6 | 1.0% |
| 5.00 |  | 14 | 2.4% |
| 5.13 |  | 11 | 1.9% |
| 5.25 |  | 7 | 1.2% |
| 5.29 |  | 1 | 0.2% |
| 5.38 |  | 14 | 2.4% |
| 5.50 |  | 12 | 2.0% |
| 5.63 |  | 18 | 3.0% |
| 5.75 |  | 20 | 3.4% |
| 5.88 |  | 13 | 2.2% |
| 6.00 |  | 21 | 3.5% |
| 6.13 |  | 8 | 1.3% |
| 6.25 |  | 18 | 3.0% |
| 6.38 |  | 21 | 3.5% |
| 6.50 |  | 8 | 1.3% |
| 6.63 |  | 14 | 2.4% |
| 6.75 |  | 18 | 3.0% |
| 6.88 |  | 9 | 1.5% |
| 7.00 |  | 8 | 1.3% |
| Missing | System | 296 | 49.8% |
|  |  |  |  |
|  |  |  |  |

| **PO** |  |  |  |
| --- | --- | --- | --- |
|  |  | N | % |
| 1.00 |  | 1 | 0.2% |
| 1.67 |  | 2 | 0.3% |
| 1.83 |  | 5 | 0.8% |
| 2.00 |  | 5 | 0.8% |
| 2.17 |  | 5 | 0.8% |
| 2.33 |  | 4 | 0.7% |
| 2.50 |  | 4 | 0.7% |
| 2.67 |  | 10 | 1.7% |
| 2.83 |  | 14 | 2.4% |
| 3.00 |  | 11 | 1.9% |
| 3.17 |  | 16 | 2.7% |
| 3.33 |  | 19 | 3.2% |
| 3.40 |  | 1 | 0.2% |
| 3.50 |  | 16 | 2.7% |
| 3.67 |  | 26 | 4.4% |
| 3.83 |  | 24 | 4.0% |
| 4.00 |  | 29 | 4.9% |
| 4.17 |  | 21 | 3.5% |
| 4.33 |  | 17 | 2.9% |
| 4.50 |  | 17 | 2.9% |
| 4.67 |  | 11 | 1.9% |
| 4.83 |  | 14 | 2.4% |
| 5.00 |  | 9 | 1.5% |
| 5.17 |  | 5 | 0.8% |
| 5.33 |  | 5 | 0.8% |
| 5.50 |  | 1 | 0.2% |
| 5.67 |  | 1 | 0.2% |
| 5.83 |  | 2 | 0.3% |
| 6.00 |  | 2 | 0.3% |
| Missing | System | 297 | 50.0% |
|  |  |  |  |
|  |  |  |  |

| **CP** |  |  |  |
| --- | --- | --- | --- |
|  |  | N | % |
| 1.00 |  | 35 | 5.9% |
| 1.25 |  | 17 | 2.9% |
| 1.50 |  | 25 | 4.2% |
| 1.75 |  | 29 | 4.9% |
| 2.00 |  | 33 | 5.6% |
| 2.25 |  | 17 | 2.9% |
| 2.50 |  | 19 | 3.2% |
| 2.75 |  | 15 | 2.5% |
| 3.00 |  | 12 | 2.0% |
| 3.25 |  | 11 | 1.9% |
| 3.50 |  | 15 | 2.5% |
| 3.75 |  | 9 | 1.5% |
| 4.00 |  | 17 | 2.9% |
| 4.25 |  | 10 | 1.7% |
| 4.50 |  | 5 | 0.8% |
| 4.75 |  | 3 | 0.5% |
| 5.00 |  | 6 | 1.0% |
| 5.25 |  | 4 | 0.7% |
| 5.50 |  | 6 | 1.0% |
| 5.75 |  | 5 | 0.8% |
| 6.25 |  | 3 | 0.5% |
| 6.75 |  | 1 | 0.2% |
| Missing | System | 297 | 50.0% |
|  |  |  |  |
|  |  |  |  |

**Histogram**

**Frequencies**

| **Notes** |  |  |
| --- | --- | --- |
| Output Created |  | 20-DEC-2024 11:54:41 |
| Comments |  |  |
| Input | Data | \\apporto.com\dfs\GWU\Users\pankratimenos_gwu\Desktop\SPSSData.sav |
|  | Active Dataset | DataSet2 |
|  | Filter | <none> |
|  | Weight | <none> |
|  | Split File | <none> |
|  | N of Rows in Working Data File | 594 |
| Missing Value Handling | Definition of Missing | User-defined missing values are treated as missing. |
|  | Cases Used | Statistics are based on all cases with valid data. |
| Syntax |  | FREQUENCIES VARIABLES=ST_C SPC_C PO_C CP_C /NTILES=4 /STATISTICS=STDDEV VARIANCE MINIMUM MAXIMUM MEAN MEDIAN /HISTOGRAM /ORDER=ANALYSIS. |
| Resources | Processor Time | 00:00:00.74 |
|  | Elapsed Time | 00:00:00.44 |

| **Statistics** |  |  |  |  |  |
| --- | --- | --- | --- | --- | --- |
|  |  | ST_C | SPC_C | PO_C | CP_C |
| N | Valid | 296 | 296 | 295 | 295 |
|  | Missing | 298 | 298 | 299 | 299 |
| Mean |  | 1.6626 | 5.2210 | 2.9444 | 2.6718 |
| Median |  | 1.2857 | 5.4375 | 3.0000 | 2.3333 |
| Std. Deviation |  | .89629 | .75244 | 1.03037 | 1.47538 |
| Variance |  | .803 | .566 | 1.062 | 2.177 |
| Minimum |  | 1.00 | 2.25 | 1.00 | 1.00 |
| Maximum |  | 6.00 | 6.00 | 6.00 | 6.00 |
| Percentiles | 25 | 1.0000 | 4.7500 | 2.0000 | 1.3333 |
|  | 50 | 1.2857 | 5.4375 | 3.0000 | 2.3333 |
|  | 75 | 1.8571 | 5.7500 | 3.8000 | 4.0000 |

**Frequency Table**

| **ST_C** |  |  |  |
| --- | --- | --- | --- |
|  |  | N | % |
| 1.00 |  | 95 | 16.0% |
| 1.14 |  | 32 | 5.4% |
| 1.29 |  | 33 | 5.6% |
| 1.43 |  | 13 | 2.2% |
| 1.57 |  | 22 | 3.7% |
| 1.71 |  | 18 | 3.0% |
| 1.86 |  | 15 | 2.5% |
| 2.00 |  | 6 | 1.0% |
| 2.14 |  | 7 | 1.2% |
| 2.29 |  | 9 | 1.5% |
| 2.43 |  | 3 | 0.5% |
| 2.57 |  | 3 | 0.5% |
| 2.71 |  | 5 | 0.8% |
| 2.86 |  | 3 | 0.5% |
| 3.00 |  | 1 | 0.2% |
| 3.14 |  | 6 | 1.0% |
| 3.29 |  | 6 | 1.0% |
| 3.43 |  | 1 | 0.2% |
| 3.57 |  | 2 | 0.3% |
| 3.71 |  | 2 | 0.3% |
| 3.86 |  | 1 | 0.2% |
| 4.00 |  | 2 | 0.3% |
| 4.14 |  | 3 | 0.5% |
| 4.29 |  | 3 | 0.5% |
| 4.43 |  | 1 | 0.2% |
| 4.71 |  | 1 | 0.2% |
| 4.86 |  | 2 | 0.3% |
| 6.00 |  | 1 | 0.2% |
| Missing | System | 298 | 50.2% |
|  |  |  |  |
|  |  |  |  |

| **SPC_C** |  |  |  |
| --- | --- | --- | --- |
|  |  | N | % |
| 2.25 |  | 1 | 0.2% |
| 2.38 |  | 1 | 0.2% |
| 2.75 |  | 1 | 0.2% |
| 2.88 |  | 2 | 0.3% |
| 3.00 |  | 1 | 0.2% |
| 3.25 |  | 1 | 0.2% |
| 3.38 |  | 2 | 0.3% |
| 3.50 |  | 1 | 0.2% |
| 3.63 |  | 2 | 0.3% |
| 3.75 |  | 4 | 0.7% |
| 3.88 |  | 3 | 0.5% |
| 4.00 |  | 7 | 1.2% |
| 4.13 |  | 4 | 0.7% |
| 4.25 |  | 8 | 1.3% |
| 4.38 |  | 9 | 1.5% |
| 4.50 |  | 9 | 1.5% |
| 4.63 |  | 10 | 1.7% |
| 4.75 |  | 10 | 1.7% |
| 4.88 |  | 6 | 1.0% |
| 5.00 |  | 18 | 3.0% |
| 5.13 |  | 10 | 1.7% |
| 5.25 |  | 15 | 2.5% |
| 5.29 |  | 1 | 0.2% |
| 5.38 |  | 22 | 3.7% |
| 5.50 |  | 27 | 4.5% |
| 5.63 |  | 21 | 3.5% |
| 5.75 |  | 28 | 4.7% |
| 5.88 |  | 21 | 3.5% |
| 6.00 |  | 51 | 8.6% |
| Missing | System | 298 | 50.2% |
|  |  |  |  |
|  |  |  |  |

| **PO_C** |  |  |  |
| --- | --- | --- | --- |
|  |  | N | % |
| 1.00 |  | 4 | 0.7% |
| 1.20 |  | 3 | 0.5% |
| 1.40 |  | 18 | 3.0% |
| 1.60 |  | 20 | 3.4% |
| 1.80 |  | 13 | 2.2% |
| 2.00 |  | 21 | 3.5% |
| 2.20 |  | 19 | 3.2% |
| 2.40 |  | 15 | 2.5% |
| 2.60 |  | 7 | 1.2% |
| 2.80 |  | 18 | 3.0% |
| 3.00 |  | 18 | 3.0% |
| 3.20 |  | 26 | 4.4% |
| 3.40 |  | 13 | 2.2% |
| 3.60 |  | 16 | 2.7% |
| 3.80 |  | 18 | 3.0% |
| 4.00 |  | 22 | 3.7% |
| 4.20 |  | 17 | 2.9% |
| 4.40 |  | 14 | 2.4% |
| 4.60 |  | 6 | 1.0% |
| 4.80 |  | 2 | 0.3% |
| 5.00 |  | 3 | 0.5% |
| 5.40 |  | 1 | 0.2% |
| 6.00 |  | 1 | 0.2% |
| Missing | System | 299 | 50.3% |
|  |  |  |  |
|  |  |  |  |

| **CP_C** |  |  |  |
| --- | --- | --- | --- |
|  |  | N | % |
| 1.00 |  | 58 | 9.8% |
| 1.17 |  | 15 | 2.5% |
| 1.33 |  | 15 | 2.5% |
| 1.50 |  | 16 | 2.7% |
| 1.67 |  | 11 | 1.9% |
| 1.83 |  | 10 | 1.7% |
| 2.00 |  | 15 | 2.5% |
| 2.17 |  | 5 | 0.8% |
| 2.33 |  | 4 | 0.7% |
| 2.50 |  | 8 | 1.3% |
| 2.67 |  | 7 | 1.2% |
| 2.83 |  | 2 | 0.3% |
| 3.00 |  | 18 | 3.0% |
| 3.17 |  | 4 | 0.7% |
| 3.33 |  | 6 | 1.0% |
| 3.50 |  | 4 | 0.7% |
| 3.67 |  | 10 | 1.7% |
| 3.83 |  | 4 | 0.7% |
| 4.00 |  | 18 | 3.0% |
| 4.17 |  | 7 | 1.2% |
| 4.33 |  | 8 | 1.3% |
| 4.50 |  | 8 | 1.3% |
| 4.67 |  | 8 | 1.3% |
| 4.83 |  | 7 | 1.2% |
| 5.00 |  | 16 | 2.7% |
| 5.17 |  | 1 | 0.2% |
| 5.33 |  | 5 | 0.8% |
| 6.00 |  | 5 | 0.8% |
| Missing | System | 299 | 50.3% |
|  |  |  |  |
|  |  |  |  |

**Histogram**
