# Supplementary material for: Cultural attitudes toward sport psychology: insights from Italian athletes and coaches
Source: Front Psychol. 2025 Aug 4;16:1630005. doi: 10.3389/fpsyg.2025.1630005 (PMC12358425; doi:10.3389/fpsyg.2025.1630005)
Supplement: Supplementary File 3 — SPSS outputs. [file Supplementary_file_3.docx]

**Reliability**

| **Notes** |  |  |
| --- | --- | --- |
| Output Created |  | 09-DEC-2024 10:58:52 |
| Comments |  |  |
| Input | Active Dataset | DataSet1 |
|  | Filter | <none> |
|  | Weight | <none> |
|  | Split File | <none> |
|  | N of Rows in Working Data File | 594 |
|  | Matrix Input |  |
| Missing Value Handling | Definition of Missing | User-defined missing values are treated as missing. |
|  | Cases Used | Statistics are based on all cases with valid data for all variables in the procedure. |
| Syntax |  | RELIABILITY /VARIABLES=Q12_2 Q12_3 Q12_5 Q12_6 Q12_7 /SCALE('ALL VARIABLES') ALL /MODEL=ALPHA /STATISTICS=DESCRIPTIVE SCALE CORR /SUMMARY=TOTAL. |
| Resources | Processor Time | 00:00:00.00 |
|  | Elapsed Time | 00:00:00.00 |

[DataSet1]

**Scale: ALL VARIABLES**

| **Case Processing Summary** |  |  |  |
| --- | --- | --- | --- |
|  |  | N | % |
| Cases | Valid | 297 | 50.0 |
|  | Excluded^a^ | 297 | 50.0 |
|  | Total | 594 | 100.0 |

| a. Listwise deletion based on all variables in the procedure. |  |  |  |
| --- | --- | --- | --- |

| **Reliability Statistics** |  |  |
| --- | --- | --- |
| Cronbach's Alpha | Cronbach's Alpha Based on Standardized Items | N of Items |
| .596 | .615 | 5 |

| **Item Statistics** |  |  |  |
| --- | --- | --- | --- |
|  | Mean | Std. Deviation | N |
| Q12_2 | 4.43 | 1.582 | 297 |
| Q12_3 | 3.51 | 1.511 | 297 |
| Q12_5 | 2.71 | 1.264 | 297 |
| Q12_6 | 2.57 | 1.543 | 297 |
| Q12_7 | 3.92 | 1.887 | 297 |

| **Inter-Item Correlation Matrix** |  |  |  |  |  |
| --- | --- | --- | --- | --- | --- |
|  | Q12_2 | Q12_3 | Q12_5 | Q12_6 | Q12_7 |
| Q12_2 | 1.000 | .275 | .284 | .141 | .055 |
| Q12_3 | .275 | 1.000 | .423 | .266 | .159 |
| Q12_5 | .284 | .423 | 1.000 | .322 | .122 |
| Q12_6 | .141 | .266 | .322 | 1.000 | .373 |
| Q12_7 | .055 | .159 | .122 | .373 | 1.000 |

| **Item-Total Statistics** |  |  |  |  |  |
| --- | --- | --- | --- | --- | --- |
|  | Scale Mean if Item Deleted | Scale Variance if Item Deleted | Corrected Item-Total Correlation | Squared Multiple Correlation | Cronbach's Alpha if Item Deleted |
| Q12_2 | 12.71 | 17.600 | .261 | .110 | .589 |
| Q12_3 | 13.63 | 16.206 | .417 | .225 | .507 |
| Q12_5 | 14.43 | 17.381 | .436 | .252 | .510 |
| Q12_6 | 14.58 | 15.826 | .437 | .228 | .495 |
| Q12_7 | 13.22 | 16.018 | .264 | .144 | .604 |

| **Scale Statistics** |  |  |  |
| --- | --- | --- | --- |
| Mean | Variance | Std. Deviation | N of Items |
| 17.14 | 23.568 | 4.855 | 5 |
