# Supplementary material for: Cultural attitudes toward sport psychology: insights from Italian athletes and coaches
Source: Front Psychol. 2025 Aug 4;16:1630005. doi: 10.3389/fpsyg.2025.1630005 (PMC12358425; doi:10.3389/fpsyg.2025.1630005)
Supplement: Supplementary Data Sheet 1 — Full R-analysis statistical output. [file Data_Sheet_1.pdf]

##Reading data

```
data <- read_excel("Perception of Sport Psychology in Italy - MNSU_February 20, 2023_09 copy.58.xlsx")
dim(data)
```

```
## [1] 594  69
```

```
data <- data %>%
  filter_at(vars(Q11_1:Q12_11), all_vars(!is.na(.)))
dim(data)
```

```
## [1] 294  69
```

```
data$Lvl_Competitvness[182] <- 3



data %>%
  mutate(Pre_Expo = ifelse(Pre_Expo == 1, "Yes", "No")) %>%
  gtsummary::tbl_summary(include = c(Pre_Expo))
```

Characteristic	N = 294[1]
Pre_Expo	102 (35%)

[1] n (%)

```
modela <- 'f1 =~ Q11_1 + Q11_2 + Q11_3 + Q11_4 + Q11_5 + Q11_6 + Q11_7
        f2 =~ Q11_8 + Q11_9 + Q11_10 + Q11_11 + Q11_12 + Q11_13 + Q11_14 + Q12_1
        f3 =~ Q12_2 + Q12_2 + Q12_2 + Q12_2 + Q12_3 + Q12_4 + Q12_5 + Q12_6 + Q12_7
        f4 =~ Q12_8 + Q12_9 + Q12_10 + Q12_11
        f3 =~ 1*f1 + 1*f2 + 1*f3 + 1*f4
        f3 ~~ f3'
secondorder <- cfa(modela, data = data)
summary(secondorder, fit.measures = TRUE, standardized = TRUE)
```

```
## lavaan 0.6-19 ended normally after 84 iterations
##
##   Estimator                                         ML
##   Optimization method                           NLMINB
##   Number of model parameters                        50
##
##   Number of observations                           294
##
## Model Test User Model:
##
##   Test statistic                               752.314
##   Degrees of freedom                               275
##   P-value (Chi-square)                           0.000
##
## Model Test Baseline Model:
##
##   Test statistic                              3501.223
##   Degrees of freedom                               300
##   P-value                                        0.000
##
## User Model versus Baseline Model:
##
##   Comparative Fit Index (CFI)                    0.851
##   Tucker-Lewis Index (TLI)                       0.837
##
## Loglikelihood and Information Criteria:
##
##   Loglikelihood user model (H0)             -11731.564
##   Loglikelihood unrestricted model (H1)     -11355.407
##
##   Akaike (AIC)                               23563.127
##   Bayesian (BIC)                             23747.306
##   Sample-size adjusted Bayesian (SABIC)      23588.743
##
## Root Mean Square Error of Approximation:
##
##   RMSEA                                          0.077
##   90 Percent confidence interval - lower         0.070
##   90 Percent confidence interval - upper         0.083
```

```
##   P-value H_0: RMSEA <= 0.050                    0.000
##   P-value H_0: RMSEA >= 0.080                    0.219
##
## Standardized Root Mean Square Residual:
##
##   SRMR                                           0.140
##
## Parameter Estimates:
##
##   Standard errors                             Standard
##   Information                                 Expected
##   Information saturated (h1) model          Structured
##
## Latent Variables:
##                    Estimate  Std.Err  z-value  P(>|z|)   Std.lv  Std.all
##   f1 =~
##     Q11_1             1.000                               1.268    0.857
##     Q11_2             0.948    0.053   17.952    0.000    1.201    0.849
##     Q11_3             0.978    0.060   16.349    0.000    1.240    0.798
##     Q11_4             0.594    0.063    9.367    0.000    0.754    0.525
##     Q11_5             0.565    0.047   12.053    0.000    0.717    0.643
##     Q11_6             0.701    0.053   13.283    0.000    0.889    0.691
##     Q11_7             0.599    0.048   12.431    0.000    0.760    0.658
##   f2 =~
##     Q11_8             1.000                               0.995    0.748
##     Q11_9             1.139    0.091   12.480    0.000    1.133    0.716
##     Q11_10            1.001    0.066   15.100    0.000    0.995    0.849
##     Q11_11            1.139    0.077   14.857    0.000    1.133    0.836
##     Q11_12            0.793    0.059   13.416    0.000    0.789    0.764
##     Q11_13            1.191    0.099   12.038    0.000    1.185    0.694
##     Q11_14            1.274    0.084   15.228    0.000    1.267    0.855
##     Q12_1             0.743    0.103    7.182    0.000    0.739    0.427
##   f3 =~
##     Q12_2             1.000                               0.241    0.156
##     Q12_3             3.369    0.732    4.601    0.000    0.813    0.538
##     Q12_4             0.267    0.436    0.612    0.541    0.064    0.044
##     Q12_5             3.145    0.663    4.743    0.000    0.759    0.603
##     Q12_6             3.926    0.825    4.758    0.000    0.948    0.614
##     Q12_7             3.070    0.765    4.012    0.000    0.741    0.392
```

```
##   f4 =~
##     Q12_8             1.000                               0.974    0.574
##     Q12_9             1.276    0.134    9.533    0.000    1.243    0.773
##     Q12_10            1.426    0.144    9.873    0.000    1.389    0.855
##     Q12_11            1.205    0.133    9.045    0.000    1.174    0.706
##   f3 =~
##     f1                1.000                               0.190    0.190
##     f2                1.000                               0.243    0.243
##     f3                1.000                               1.000    1.000
##     f4                1.000                               0.248    0.248
##
## Variances:
##                    Estimate  Std.Err  z-value  P(>|z|)   Std.lv  Std.all
##    .f3                0.058    0.021    2.752    0.006    1.000    1.000
##    .Q11_1             0.584    0.068    8.629    0.000    0.584    0.266
##    .Q11_2             0.560    0.063    8.847    0.000    0.560    0.279
##    .Q11_3             0.876    0.088    9.911    0.000    0.876    0.363
##    .Q11_4             1.492    0.128   11.654    0.000    1.492    0.724
##    .Q11_5             0.728    0.065   11.250    0.000    0.728    0.586
##    .Q11_6             0.863    0.079   10.985    0.000    0.863    0.522
##    .Q11_7             0.755    0.068   11.175    0.000    0.755    0.567
##    .Q11_8             0.779    0.072   10.884    0.000    0.779    0.441
##    .Q11_9             1.218    0.110   11.095    0.000    1.218    0.487
##    .Q11_10            0.385    0.040    9.588    0.000    0.385    0.280
##    .Q11_11            0.551    0.056    9.829    0.000    0.551    0.300
##    .Q11_12            0.443    0.041   10.749    0.000    0.443    0.416
##    .Q11_13            1.514    0.135   11.221    0.000    1.514    0.519
##    .Q11_14            0.591    0.063    9.445    0.000    0.591    0.269
##    .Q12_1             2.444    0.205   11.908    0.000    2.444    0.818
##    .Q12_2             2.332    0.194   12.013    0.000    2.332    0.976
##    .Q12_3             1.623    0.171    9.469    0.000    1.623    0.710
##    .Q12_4             2.122    0.175   12.112    0.000    2.122    0.998
##    .Q12_5             1.007    0.121    8.356    0.000    1.007    0.636
##    .Q12_6             1.482    0.182    8.138    0.000    1.482    0.623
##    .Q12_7             3.017    0.275   10.967    0.000    3.017    0.846
##    .Q12_8             1.929    0.175   11.052    0.000    1.929    0.670
##    .Q12_9             1.037    0.122    8.515    0.000    1.037    0.402
##    .Q12_10            0.712    0.120    5.955    0.000    0.712    0.269
##    .Q12_11            1.382    0.141    9.835    0.000    1.382    0.501
```

```
##    .f1                1.550    0.176    8.785    0.000    0.964    0.964
##    .f2                0.931    0.129    7.236    0.000    0.941    0.941
##    .f4                0.891    0.178    4.991    0.000    0.939    0.939
```

```
paramEsts <- parameterEstimates(secondorder)
paramEsts
```

```
##       lhs op    rhs   est    se      z pvalue ci.lower ci.upper
## 1      f1 =~  Q11_1 1.000 0.000     NA     NA    1.000    1.000
## 2      f1 =~  Q11_2 0.948 0.053 17.952  0.000    0.844    1.051
## 3      f1 =~  Q11_3 0.978 0.060 16.349  0.000    0.861    1.095
## 4      f1 =~  Q11_4 0.594 0.063  9.367  0.000    0.470    0.719
## 5      f1 =~  Q11_5 0.565 0.047 12.053  0.000    0.473    0.657
## 6      f1 =~  Q11_6 0.701 0.053 13.283  0.000    0.598    0.805
## 7      f1 =~  Q11_7 0.599 0.048 12.431  0.000    0.505    0.694
## 8      f2 =~  Q11_8 1.000 0.000     NA     NA    1.000    1.000
## 9      f2 =~  Q11_9 1.139 0.091 12.480  0.000    0.960    1.318
## 10     f2 =~ Q11_10 1.001 0.066 15.100  0.000    0.871    1.131
## 11     f2 =~ Q11_11 1.139 0.077 14.857  0.000    0.989    1.290
## 12     f2 =~ Q11_12 0.793 0.059 13.416  0.000    0.677    0.909
## 13     f2 =~ Q11_13 1.191 0.099 12.038  0.000    0.997    1.385
## 14     f2 =~ Q11_14 1.274 0.084 15.228  0.000    1.110    1.438
## 15     f2 =~  Q12_1 0.743 0.103  7.182  0.000    0.540    0.945
## 16     f3 =~  Q12_2 1.000 0.000     NA     NA    1.000    1.000
## 17     f3 =~  Q12_3 3.369 0.732  4.601  0.000    1.934    4.804
## 18     f3 =~  Q12_4 0.267 0.436  0.612  0.541   -0.587    1.121
## 19     f3 =~  Q12_5 3.145 0.663  4.743  0.000    1.845    4.445
## 20     f3 =~  Q12_6 3.926 0.825  4.758  0.000    2.309    5.543
## 21     f3 =~  Q12_7 3.070 0.765  4.012  0.000    1.570    4.570
## 22     f4 =~  Q12_8 1.000 0.000     NA     NA    1.000    1.000
## 23     f4 =~  Q12_9 1.276 0.134  9.533  0.000    1.013    1.538
## 24     f4 =~ Q12_10 1.426 0.144  9.873  0.000    1.143    1.709
## 25     f4 =~ Q12_11 1.205 0.133  9.045  0.000    0.944    1.466
## 26     f3 =~     f1 1.000 0.000     NA     NA    1.000    1.000
## 27     f3 =~     f2 1.000 0.000     NA     NA    1.000    1.000
## 28     f3 =~     f3 1.000 0.000     NA     NA    1.000    1.000
## 29     f3 =~     f4 1.000 0.000     NA     NA    1.000    1.000
## 30     f3 ~~     f3 0.058 0.021  2.752  0.006    0.017    0.100
## 31  Q11_1 ~~  Q11_1 0.584 0.068  8.629  0.000    0.451    0.716
## 32  Q11_2 ~~  Q11_2 0.560 0.063  8.847  0.000    0.436    0.684
## 33  Q11_3 ~~  Q11_3 0.876 0.088  9.911  0.000    0.703    1.050
## 34  Q11_4 ~~  Q11_4 1.492 0.128 11.654  0.000    1.241    1.742
## 35  Q11_5 ~~  Q11_5 0.728 0.065 11.250  0.000    0.601    0.855
## 36  Q11_6 ~~  Q11_6 0.863 0.079 10.985  0.000    0.709    1.017
## 37  Q11_7 ~~  Q11_7 0.755 0.068 11.175  0.000    0.622    0.887
## 38  Q11_8 ~~  Q11_8 0.779 0.072 10.884  0.000    0.639    0.919
```

```
## 39  Q11_9 ~~  Q11_9 1.218 0.110 11.095  0.000    1.003    1.433
## 40 Q11_10 ~~ Q11_10 0.385 0.040  9.588  0.000    0.306    0.464
## 41 Q11_11 ~~ Q11_11 0.551 0.056  9.829  0.000    0.441    0.661
## 42 Q11_12 ~~ Q11_12 0.443 0.041 10.749  0.000    0.362    0.524
## 43 Q11_13 ~~ Q11_13 1.514 0.135 11.221  0.000    1.250    1.779
## 44 Q11_14 ~~ Q11_14 0.591 0.063  9.445  0.000    0.469    0.714
## 45  Q12_1 ~~  Q12_1 2.444 0.205 11.908  0.000    2.042    2.847
## 46  Q12_2 ~~  Q12_2 2.332 0.194 12.013  0.000    1.952    2.713
## 47  Q12_3 ~~  Q12_3 1.623 0.171  9.469  0.000    1.287    1.958
## 48  Q12_4 ~~  Q12_4 2.122 0.175 12.112  0.000    1.778    2.465
## 49  Q12_5 ~~  Q12_5 1.007 0.121  8.356  0.000    0.771    1.244
## 50  Q12_6 ~~  Q12_6 1.482 0.182  8.138  0.000    1.125    1.838
## 51  Q12_7 ~~  Q12_7 3.017 0.275 10.967  0.000    2.478    3.557
## 52  Q12_8 ~~  Q12_8 1.929 0.175 11.052  0.000    1.587    2.271
## 53  Q12_9 ~~  Q12_9 1.037 0.122  8.515  0.000    0.798    1.276
## 54 Q12_10 ~~ Q12_10 0.712 0.120  5.955  0.000    0.478    0.947
## 55 Q12_11 ~~ Q12_11 1.382 0.141  9.835  0.000    1.107    1.658
## 56     f1 ~~     f1 1.550 0.176  8.785  0.000    1.204    1.895
## 57     f2 ~~     f2 0.931 0.129  7.236  0.000    0.679    1.183
## 58     f4 ~~     f4 0.891 0.178  4.991  0.000    0.541    1.241
```

```
fscores_athlete <- lavPredict(secondorder)
head(fscores_athlete)
```

```
##              f1         f2          f3         f4
## [1,] -1.0416508 -0.2075447 -0.04562830 -1.0060894
## [2,]  1.1537002  0.5694837 -0.08180354 -0.4622162
## [3,] -0.8390207  0.3265766  0.21804385 -0.1420861
## [4,] -0.7873737  0.5834844 -0.31169090 -0.8527551
## [5,]  2.2875358 -0.5148860  0.12811420 -0.7833746
## [6,]  0.6456768  0.7530060 -0.02968816  0.6637092
```

```
data <- cbind(data, fscores_athlete)

fscores_athlete <- data.frame(fscores_athlete)
library(tidyverse)
```

```
## ── Attaching core tidyverse packages ──────────────────────── tidyverse 2.0.0 ──
## ✔ lubridate 1.9.4     ✔ tibble    3.2.1
## ✔ readr     2.1.5     ✔ tidyr     1.3.1
## ✔ stringr   1.5.1
## ── Conflicts ────────────────────────────────────────── tidyverse_conflicts() ──
## ✖ dplyr::filter() masks stats::filter()
## ✖ dplyr::lag()    masks stats::lag()
## ℹ Use the conflicted package (<http://conflicted.r-lib.org/>) to force all conflicts to become errors
```

```
fscores_athlete %>%
  pivot_longer(everything()) %>%
  ggplot(aes(x = name,y = value,fill = name))+
  geom_boxplot()+
  facet_wrap(.~name,scale='free') +
  theme_light()
```

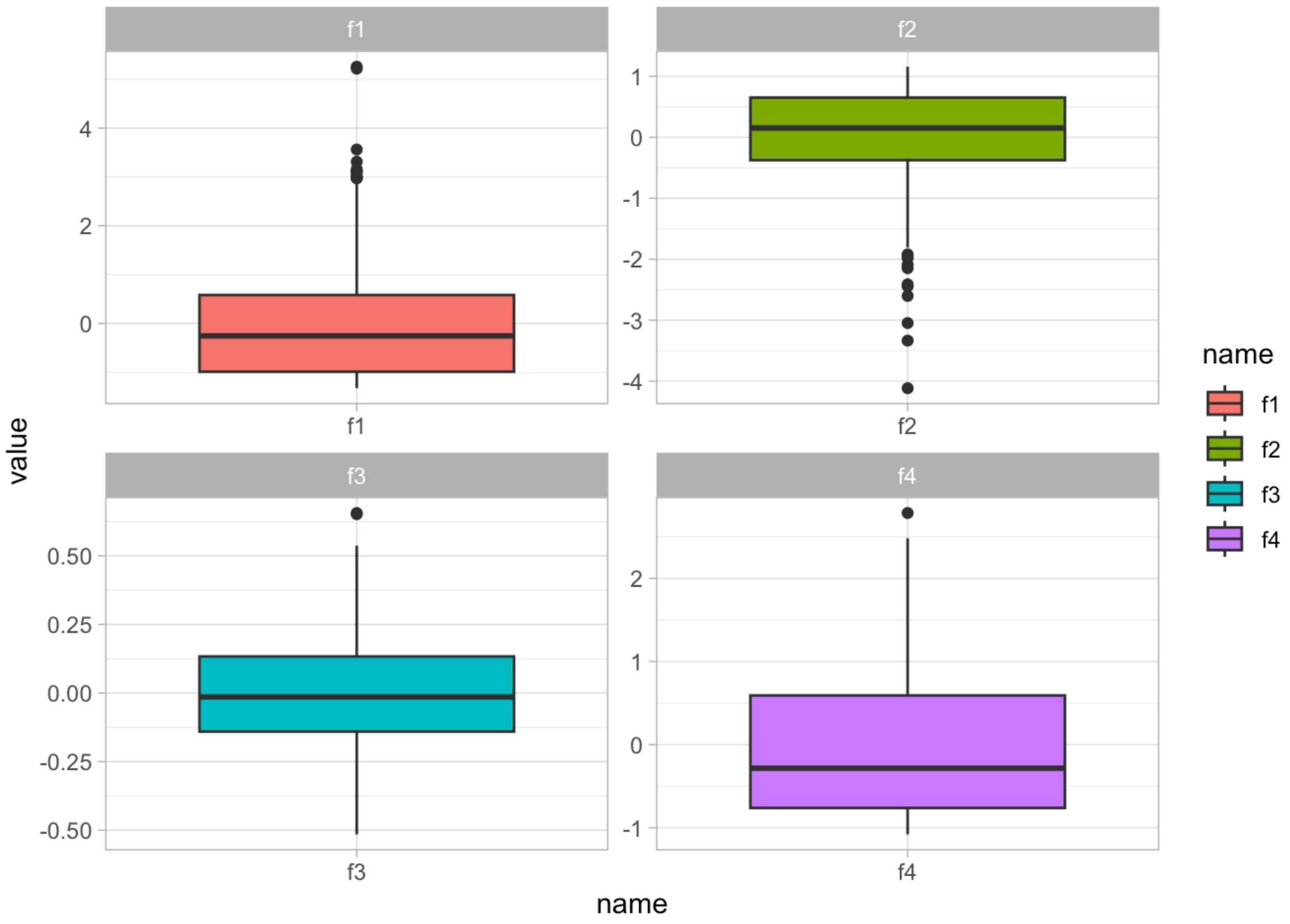


```
### Gender vs f1----f4
## testing gender and f1-f4
table(data$Gender)
```

```
##
##   1   2
## 141 153
```

```r
t1 <- t.test(f1 ~ Gender, data)
t2 <- t.test(f2 ~ Gender, data)
t3 <- t.test(f3 ~ Gender, data)
t4 <- t.test(f4 ~ Gender, data)
tab <- map_df(list(t1, t2, t3, t4), tidy)
tab
```

```
## # A tibble: 4 × 10
##   estimate estimate1 estimate2 statistic p.value parameter conf.low conf.high
##      <dbl>     <dbl>     <dbl>     <dbl>   <dbl>     <dbl>    <dbl>     <dbl>
## 1   0.285     0.148    -0.137       1.93 0.0546       289. -0.00565    0.576
## 2  -0.132    -0.0688    0.0634     -1.29 0.197        284. -0.334      0.0691
## 3   0.0612    0.0319   -0.0294      2.69 0.00766      282.  0.0164     0.106
## 4   0.218     0.114    -0.105       2.05 0.0417       274.  0.00823    0.428
## # ℹ 2 more variables: method <chr>, alternative <chr>
```

```r
data <- data %>%
  mutate(Gender = recode_factor(Gender, `1` = "Male",
                      `2` = "Female"))

p1 <- ggplot(data, aes(Gender, f1)) + geom_boxplot()
p2 <- ggplot(data, aes(Gender, f2)) + geom_boxplot()
p3 <- ggplot(data, aes(Gender, f3)) + geom_boxplot()
p4 <- ggplot(data, aes(Gender, f4)) + geom_boxplot()

ggpubr::ggarrange(p1, p2, p3, p4)
```

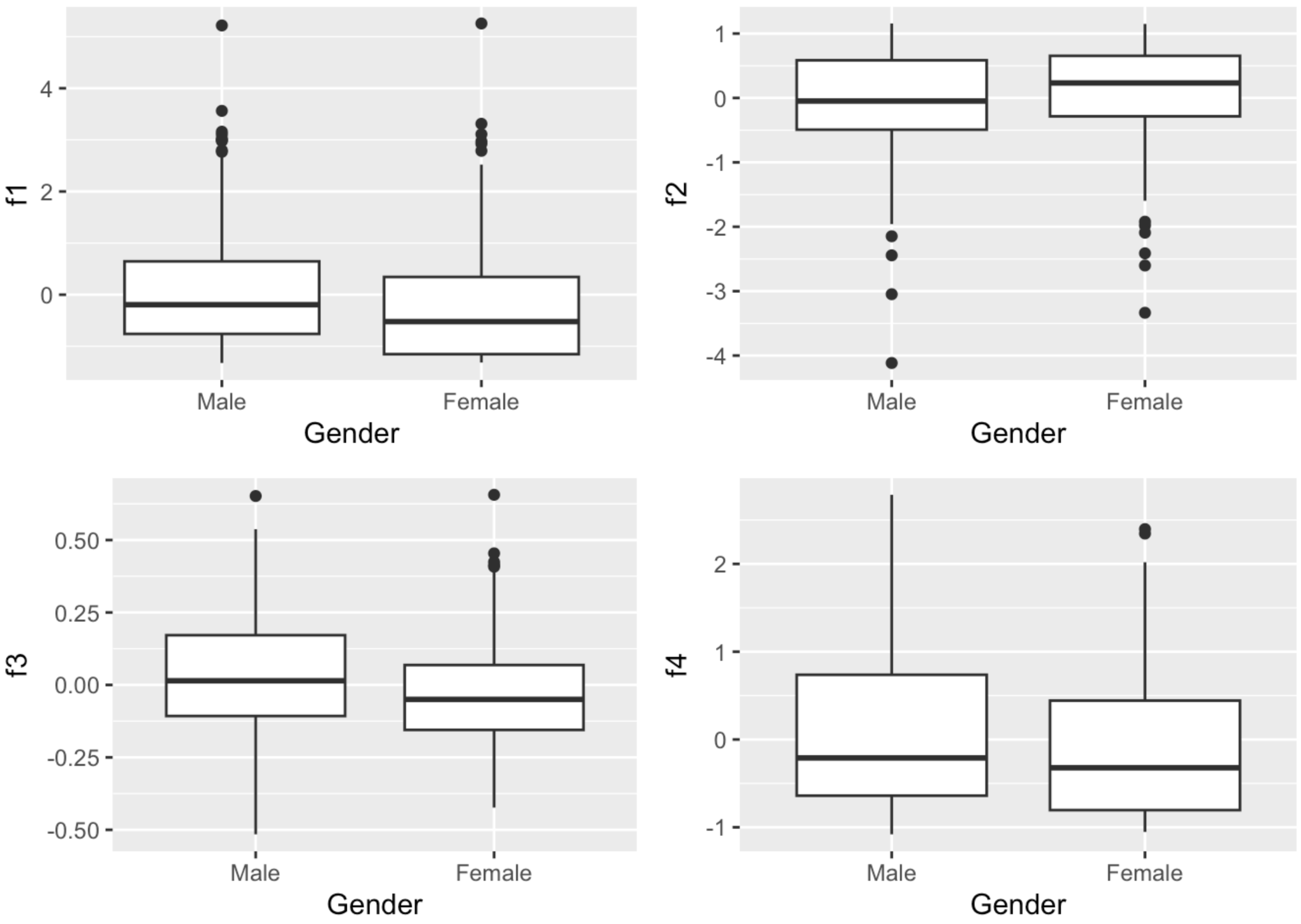


```
### Age vs f1----f4
## testing age and f1-f4

t1 <- lm(f1 ~ Age, data)
t2 <- lm(f2 ~ Age, data)
t3 <- lm(f3 ~ Age, data)
t4 <- lm(f4 ~ Age, data)
tab <- map_df(list(t1, t2, t3, t4), tidy)
tab
```

```
## # A tibble: 8 × 5
##   term        estimate std.error statistic p.value
##   <chr>          <dbl>     <dbl>     <dbl>   <dbl>
## 1 (Intercept) -0.0462     0.102     -0.453   0.651
## 2 Age          0.00624    0.00943    0.661   0.509
## 3 (Intercept) -0.0612     0.0700    -0.874   0.383
## 4 Age          0.00826    0.00648    1.28    0.203
## 5 (Intercept) -0.00249    0.0158    -0.158   0.874
## 6 Age          0.000337   0.00146    0.231   0.818
## 7 (Intercept) -0.0738     0.0729    -1.01    0.312
## 8 Age          0.00997    0.00675    1.48    0.141
```

```
p1 <- ggplot(data, aes(Age, f1)) + geom_point() + theme_light()
p2 <- ggplot(data, aes(Age, f2)) + geom_point() + theme_light()
p3 <- ggplot(data, aes(Age, f3)) + geom_point() + theme_light()
p4 <- ggplot(data, aes(Age, f4)) + geom_point() + theme_light()

ggpubr::ggarrange(p1, p2, p3, p4)
```

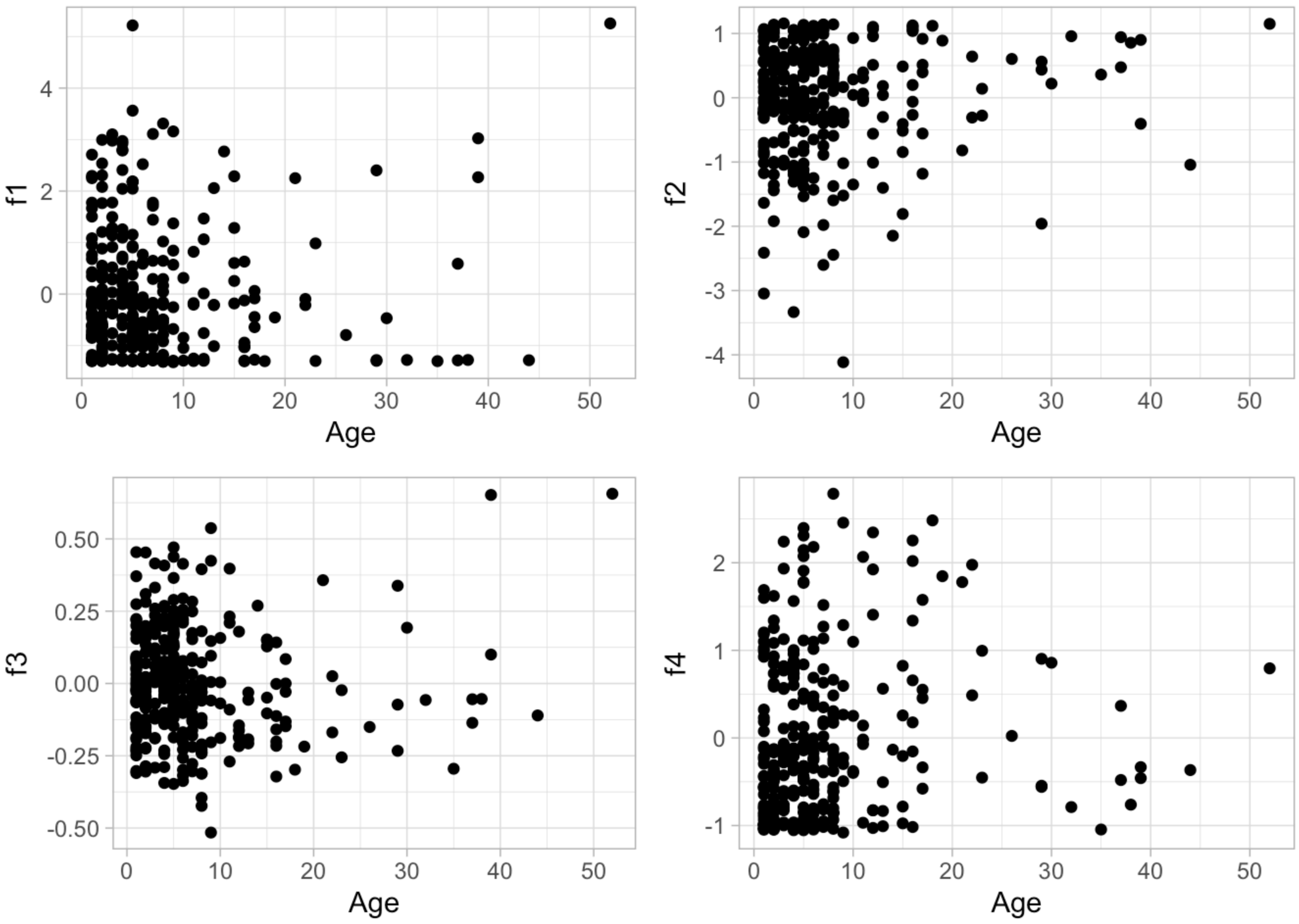


```
### lvl_competitiveness vs f1----f4
## testing lvl_competitiveness and f1-f4

t1 <- anova(lm(f1 ~ Lvl_Competitvness, data))
t2 <- anova(lm(f2 ~ Lvl_Competitvness, data))
t3 <- anova(lm(f3 ~ Lvl_Competitvness, data))
t4 <- anova(lm(f4 ~ Lvl_Competitvness, data))
tab <- map_df(list(t1, t2, t3, t4), tidy)
tab
```

```
## # A tibble: 8 × 6
##   term                 df      sumsq   meansq statistic p.value
##   <chr>             <int>      <dbl>    <dbl>     <dbl>   <dbl>
## 1 Lvl_Competitvness     1   5.76     5.76        3.60    0.0586
## 2 Residuals           292 467.       1.60       NA      NA
## 3 Lvl_Competitvness     1   3.55     3.55        4.71    0.0309
## 4 Residuals           292 221.       0.755      NA      NA
## 5 Lvl_Competitvness     1   0.000870 0.000870    0.0224  0.881
## 6 Residuals           292  11.3      0.0387     NA      NA
## 7 Lvl_Competitvness     1   1.79     1.79        2.16    0.143
## 8 Residuals           292 242.       0.829      NA      NA
```

```
data <- data %>%
  mutate(Lvl_Competitvness = recode_factor(Lvl_Competitvness,
                              `1` = "Provincial",
                              `2` = "Regional",
                              `3` = "National",
                              `4` = "International"))



p1 <- ggplot(data, aes(Lvl_Competitvness, f1)) + geom_boxplot() + theme_light()
p2 <- ggplot(data, aes(Lvl_Competitvness, f2)) + geom_boxplot() + theme_light()
p3 <- ggplot(data, aes(Lvl_Competitvness, f3)) + geom_boxplot() + theme_light()
p4 <- ggplot(data, aes(Lvl_Competitvness, f4)) + geom_boxplot() + theme_light()

ggpubr::ggarrange(p1, p2, p3, p4)
```

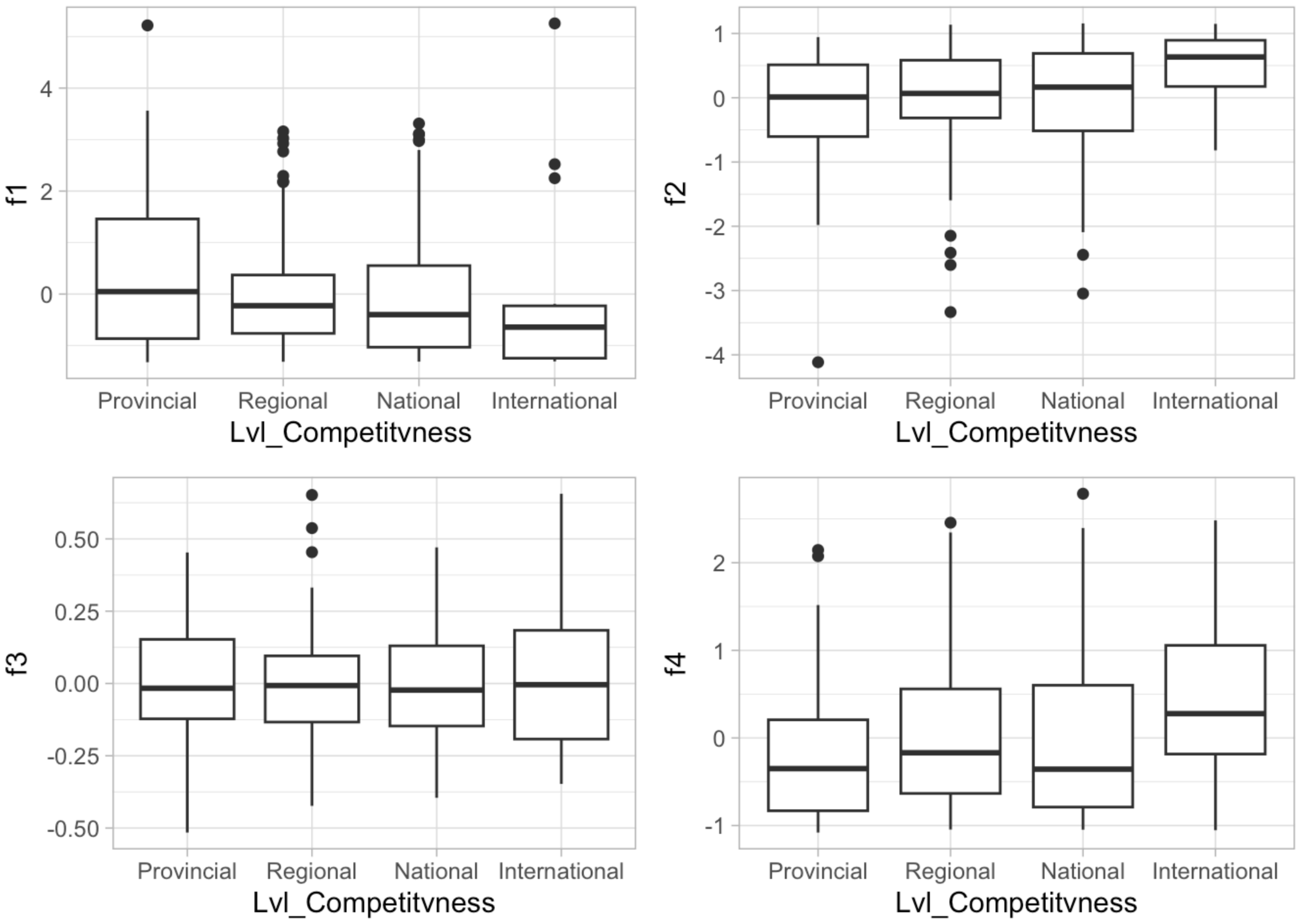


```
table(data$Lvl_Competitvness, data$Gender)
```

```
##
##                    Male Female
##   Provincial      21     15
##   Regional        40     54
##   National        71     74
##   International    9     10
```

```
data <- read_excel("Perception of Sport Psychology in Italy - MNSU_February 20, 2023_09 copy.58.xlsx")
dim(data)
```

```
## [1] 594  69
```

```
data <- data %>%
  filter_at(vars(Q13_1:Q14_12), all_vars(!is.na(.)))
dim(data)
```

```
## [1] 293  69
```

```
modelb <- 'f1 =~ Q13_1 + Q13_2 + Q13_3 + Q13_4 + Q13_5 + Q13_6 + Q14_5
        f2 =~ Q13_7 + Q13_8 + Q13_9 + Q13_10 + Q13_11 + Q13_12 + Q13_13 + Q13_14
        f3 =~ Q14_1 + Q14_2 + Q14_3 + Q14_4 + Q14_6
        f4 =~ Q14_7 + Q14_8 + Q14_9 + Q14_10 + Q14_11 + Q14_12'



data %>%
  mutate(Pre_Expo = ifelse(Pre_Expo == 1, "Yes", "No")) %>%
  gtsummary::tbl_summary(include = c(Pre_Expo))
```

**Characteristic**	**N = 293**[1]
Pre_Expo	162 (55%)

[1] n (%)

```
secondorder <- cfa(modelb, data = data)
summary(secondorder, fit.measures = TRUE, standardized = TRUE)
```

```
## lavaan 0.6-19 ended normally after 51 iterations
##
##   Estimator                                         ML
##   Optimization method                           NLMINB
##   Number of model parameters                        58
##
##   Number of observations                           293
##
## Model Test User Model:
##
##   Test statistic                               749.943
##   Degrees of freedom                               293
##   P-value (Chi-square)                           0.000
##
## Model Test Baseline Model:
##
##   Test statistic                              4815.195
##   Degrees of freedom                               325
##   P-value                                        0.000
##
## User Model versus Baseline Model:
##
##   Comparative Fit Index (CFI)                    0.898
##   Tucker-Lewis Index (TLI)                       0.887
##
## Loglikelihood and Information Criteria:
##
##   Loglikelihood user model (H0)             -10598.468
##   Loglikelihood unrestricted model (H1)     -10223.497
##
##   Akaike (AIC)                               21312.937
##   Bayesian (BIC)                             21526.387
##   Sample-size adjusted Bayesian (SABIC)      21342.454
##
## Root Mean Square Error of Approximation:
##
##   RMSEA                                          0.073
##   90 Percent confidence interval - lower         0.067
##   90 Percent confidence interval - upper         0.079
```

```
##   P-value H_0: RMSEA <= 0.050                    0.000
##   P-value H_0: RMSEA >= 0.080                    0.037
##
## Standardized Root Mean Square Residual:
##
##   SRMR                                           0.074
##
## Parameter Estimates:
##
##   Standard errors                             Standard
##   Information                                 Expected
##   Information saturated (h1) model          Structured
##
## Latent Variables:
##                    Estimate  Std.Err  z-value  P(>|z|)   Std.lv  Std.all
##   f1 =~
##     Q13_1             1.000                               0.980    0.750
##     Q13_2             1.052    0.075   14.083    0.000    1.030    0.817
##     Q13_3             0.917    0.069   13.354    0.000    0.898    0.778
##     Q13_4             0.871    0.069   12.619    0.000    0.853    0.739
##     Q13_5             0.616    0.051   11.979    0.000    0.604    0.704
##     Q13_6             0.955    0.070   13.728    0.000    0.936    0.798
##     Q14_5             0.618    0.079    7.834    0.000    0.606    0.472
##   f2 =~
##     Q13_7             1.000                               0.618    0.597
##     Q13_8             1.398    0.148    9.473    0.000    0.864    0.710
##     Q13_9             1.248    0.130    9.580    0.000    0.772    0.722
##     Q13_10            1.099    0.113    9.743    0.000    0.679    0.741
##     Q13_11            0.948    0.102    9.287    0.000    0.586    0.690
##     Q13_12            1.217    0.143    8.513    0.000    0.752    0.612
##     Q13_13            1.118    0.118    9.472    0.000    0.691    0.710
##     Q13_14            1.057    0.126    8.400    0.000    0.653    0.601
##   f3 =~
##     Q14_1             1.000                               0.770    0.480
##     Q14_2             1.630    0.223    7.302    0.000    1.254    0.745
##     Q14_3             1.229    0.171    7.176    0.000    0.946    0.709
##     Q14_4             1.257    0.179    7.005    0.000    0.968    0.669
##     Q14_6             0.690    0.126    5.483    0.000    0.531    0.433
##   f4 =~
```

```
##     Q14_7             1.000                               1.389    0.862
##     Q14_8             0.929    0.054   17.257    0.000    1.290    0.791
##     Q14_9             1.118    0.047   23.851    0.000    1.554    0.935
##     Q14_10            1.133    0.046   24.477    0.000    1.574    0.946
##     Q14_11            1.107    0.048   22.827    0.000    1.538    0.917
##     Q14_12            0.909    0.056   16.138    0.000    1.263    0.759
##
## Covariances:
##                    Estimate  Std.Err  z-value  P(>|z|)   Std.lv  Std.all
##   f1 ~~
##     f2               -0.292    0.052   -5.656    0.000   -0.482   -0.482
##     f3                0.330    0.070    4.722    0.000    0.437    0.437
##     f4                0.272    0.088    3.078    0.002    0.200    0.200
##   f2 ~~
##     f3               -0.220    0.048   -4.624    0.000   -0.463   -0.463
##     f4                0.058    0.055    1.058    0.290    0.068    0.068
##   f3 ~~
##     f4               -0.359    0.087   -4.144    0.000   -0.335   -0.335
##
## Variances:
##                    Estimate  Std.Err  z-value  P(>|z|)   Std.lv  Std.all
##    .Q13_1             0.749    0.072   10.464    0.000    0.749    0.438
##    .Q13_2             0.528    0.055    9.522    0.000    0.528    0.332
##    .Q13_3             0.526    0.052   10.138    0.000    0.526    0.395
##    .Q13_4             0.607    0.057   10.570    0.000    0.607    0.454
##    .Q13_5             0.371    0.034   10.850    0.000    0.371    0.504
##    .Q13_6             0.499    0.051    9.852    0.000    0.499    0.363
##    .Q14_5             1.282    0.109   11.742    0.000    1.282    0.778
##    .Q13_7             0.691    0.062   11.190    0.000    0.691    0.644
##    .Q13_8             0.733    0.070   10.412    0.000    0.733    0.495
##    .Q13_9             0.546    0.053   10.291    0.000    0.546    0.478
##    .Q13_10            0.379    0.038   10.079    0.000    0.379    0.451
##    .Q13_11            0.378    0.036   10.594    0.000    0.378    0.524
##    .Q13_12            0.947    0.085   11.114    0.000    0.947    0.626
##    .Q13_13            0.469    0.045   10.412    0.000    0.469    0.495
##    .Q13_14            0.754    0.068   11.169    0.000    0.754    0.639
##    .Q14_1             1.984    0.176   11.242    0.000    1.984    0.770
##    .Q14_2             1.261    0.150    8.412    0.000    1.261    0.445
##    .Q14_3             0.884    0.097    9.106    0.000    0.884    0.497
```

```
##    .Q14_4           1.158    0.119    9.724    0.000    1.158    0.553
##    .Q14_6           1.222    0.107   11.439    0.000    1.222    0.812
##    .Q14_7           0.665    0.062   10.773    0.000    0.665    0.256
##    .Q14_8           0.995    0.088   11.343    0.000    0.995    0.374
##    .Q14_9           0.347    0.039    8.827    0.000    0.347    0.126
##    .Q14_10          0.291    0.036    8.112    0.000    0.291    0.105
##    .Q14_11          0.449    0.047    9.651    0.000    0.449    0.160
##    .Q14_12          1.171    0.102   11.486    0.000    1.171    0.423
##     f1              0.960    0.132    7.278    0.000    1.000    1.000
##     f2              0.382    0.072    5.319    0.000    1.000    1.000
##     f3              0.592    0.151    3.931    0.000    1.000    1.000
##     f4              1.931    0.209    9.229    0.000    1.000    1.000
```

```
paramEsts <- parameterEstimates(secondorder)
paramEsts
```

```
##       lhs op    rhs   est    se      z pvalue ci.lower ci.upper
## 1      f1 =~  Q13_1 1.000 0.000     NA     NA    1.000    1.000
## 2      f1 =~  Q13_2 1.052 0.075 14.083  0.000    0.905    1.198
## 3      f1 =~  Q13_3 0.917 0.069 13.354  0.000    0.782    1.051
## 4      f1 =~  Q13_4 0.871 0.069 12.619  0.000    0.736    1.006
## 5      f1 =~  Q13_5 0.616 0.051 11.979  0.000    0.515    0.717
## 6      f1 =~  Q13_6 0.955 0.070 13.728  0.000    0.819    1.092
## 7      f1 =~  Q14_5 0.618 0.079  7.834  0.000    0.464    0.773
## 8      f2 =~  Q13_7 1.000 0.000     NA     NA    1.000    1.000
## 9      f2 =~  Q13_8 1.398 0.148  9.473  0.000    1.109    1.687
## 10     f2 =~  Q13_9 1.248 0.130  9.580  0.000    0.993    1.504
## 11     f2 =~ Q13_10 1.099 0.113  9.743  0.000    0.878    1.320
## 12     f2 =~ Q13_11 0.948 0.102  9.287  0.000    0.748    1.148
## 13     f2 =~ Q13_12 1.217 0.143  8.513  0.000    0.937    1.498
## 14     f2 =~ Q13_13 1.118 0.118  9.472  0.000    0.887    1.349
## 15     f2 =~ Q13_14 1.057 0.126  8.400  0.000    0.810    1.303
## 16     f3 =~  Q14_1 1.000 0.000     NA     NA    1.000    1.000
## 17     f3 =~  Q14_2 1.630 0.223  7.302  0.000    1.192    2.067
## 18     f3 =~  Q14_3 1.229 0.171  7.176  0.000    0.893    1.565
## 19     f3 =~  Q14_4 1.257 0.179  7.005  0.000    0.905    1.609
## 20     f3 =~  Q14_6 0.690 0.126  5.483  0.000    0.443    0.937
## 21     f4 =~  Q14_7 1.000 0.000     NA     NA    1.000    1.000
## 22     f4 =~  Q14_8 0.929 0.054 17.257  0.000    0.823    1.034
## 23     f4 =~  Q14_9 1.118 0.047 23.851  0.000    1.026    1.210
## 24     f4 =~ Q14_10 1.133 0.046 24.477  0.000    1.042    1.224
## 25     f4 =~ Q14_11 1.107 0.048 22.827  0.000    1.012    1.202
## 26     f4 =~ Q14_12 0.909 0.056 16.138  0.000    0.799    1.020
## 27  Q13_1 ~~  Q13_1 0.749 0.072 10.464  0.000    0.608    0.889
## 28  Q13_2 ~~  Q13_2 0.528 0.055  9.522  0.000    0.420    0.637
## 29  Q13_3 ~~  Q13_3 0.526 0.052 10.138  0.000    0.424    0.628
## 30  Q13_4 ~~  Q13_4 0.607 0.057 10.570  0.000    0.494    0.719
## 31  Q13_5 ~~  Q13_5 0.371 0.034 10.850  0.000    0.304    0.438
## 32  Q13_6 ~~  Q13_6 0.499 0.051  9.852  0.000    0.400    0.598
## 33  Q14_5 ~~  Q14_5 1.282 0.109 11.742  0.000    1.068    1.496
## 34  Q13_7 ~~  Q13_7 0.691 0.062 11.190  0.000    0.570    0.812
## 35  Q13_8 ~~  Q13_8 0.733 0.070 10.412  0.000    0.595    0.871
## 36  Q13_9 ~~  Q13_9 0.546 0.053 10.291  0.000    0.442    0.650
## 37 Q13_10 ~~ Q13_10 0.379 0.038 10.079  0.000    0.305    0.453
## 38 Q13_11 ~~ Q13_11 0.378 0.036 10.594  0.000    0.308    0.447
```

```
## 39 Q13_12 ~~ Q13_12  0.947 0.085 11.114  0.000    0.780    1.114
## 40 Q13_13 ~~ Q13_13  0.469 0.045 10.412  0.000    0.380    0.557
## 41 Q13_14 ~~ Q13_14  0.754 0.068 11.169  0.000    0.622    0.887
## 42  Q14_1 ~~  Q14_1  1.984 0.176 11.242  0.000    1.638    2.330
## 43  Q14_2 ~~  Q14_2  1.261 0.150  8.412  0.000    0.967    1.554
## 44  Q14_3 ~~  Q14_3  0.884 0.097  9.106  0.000    0.694    1.074
## 45  Q14_4 ~~  Q14_4  1.158 0.119  9.724  0.000    0.925    1.391
## 46  Q14_6 ~~  Q14_6  1.222 0.107 11.439  0.000    1.012    1.431
## 47  Q14_7 ~~  Q14_7  0.665 0.062 10.773  0.000    0.544    0.786
## 48  Q14_8 ~~  Q14_8  0.995 0.088 11.343  0.000    0.823    1.167
## 49  Q14_9 ~~  Q14_9  0.347 0.039  8.827  0.000    0.270    0.424
## 50 Q14_10 ~~ Q14_10  0.291 0.036  8.112  0.000    0.221    0.361
## 51 Q14_11 ~~ Q14_11  0.449 0.047  9.651  0.000    0.358    0.541
## 52 Q14_12 ~~ Q14_12  1.171 0.102 11.486  0.000    0.971    1.371
## 53     f1 ~~     f1  0.960 0.132  7.278  0.000    0.701    1.218
## 54     f2 ~~     f2  0.382 0.072  5.319  0.000    0.241    0.523
## 55     f3 ~~     f3  0.592 0.151  3.931  0.000    0.297    0.888
## 56     f4 ~~     f4  1.931 0.209  9.229  0.000    1.521    2.341
## 57     f1 ~~     f2 -0.292 0.052 -5.656  0.000   -0.393   -0.191
## 58     f1 ~~     f3  0.330 0.070  4.722  0.000    0.193    0.467
## 59     f1 ~~     f4  0.272 0.088  3.078  0.002    0.099    0.445
## 60     f2 ~~     f3 -0.220 0.048 -4.624  0.000   -0.314   -0.127
## 61     f2 ~~     f4  0.058 0.055  1.058  0.290   -0.050    0.166
## 62     f3 ~~     f4 -0.359 0.087 -4.144  0.000   -0.528   -0.189
```

```
fscores_coach <- lavPredict(secondorder)

data <- cbind(data, fscores_coach)

fscores_coach <- data.frame(fscores_coach)
library(tidyverse)
fscores_coach %>%
  pivot_longer(everything()) %>%
  ggplot(aes(x = name,y = value,fill = name))+
  geom_boxplot()+
  facet_wrap(.~name,scale='free') +
  theme_light()
```

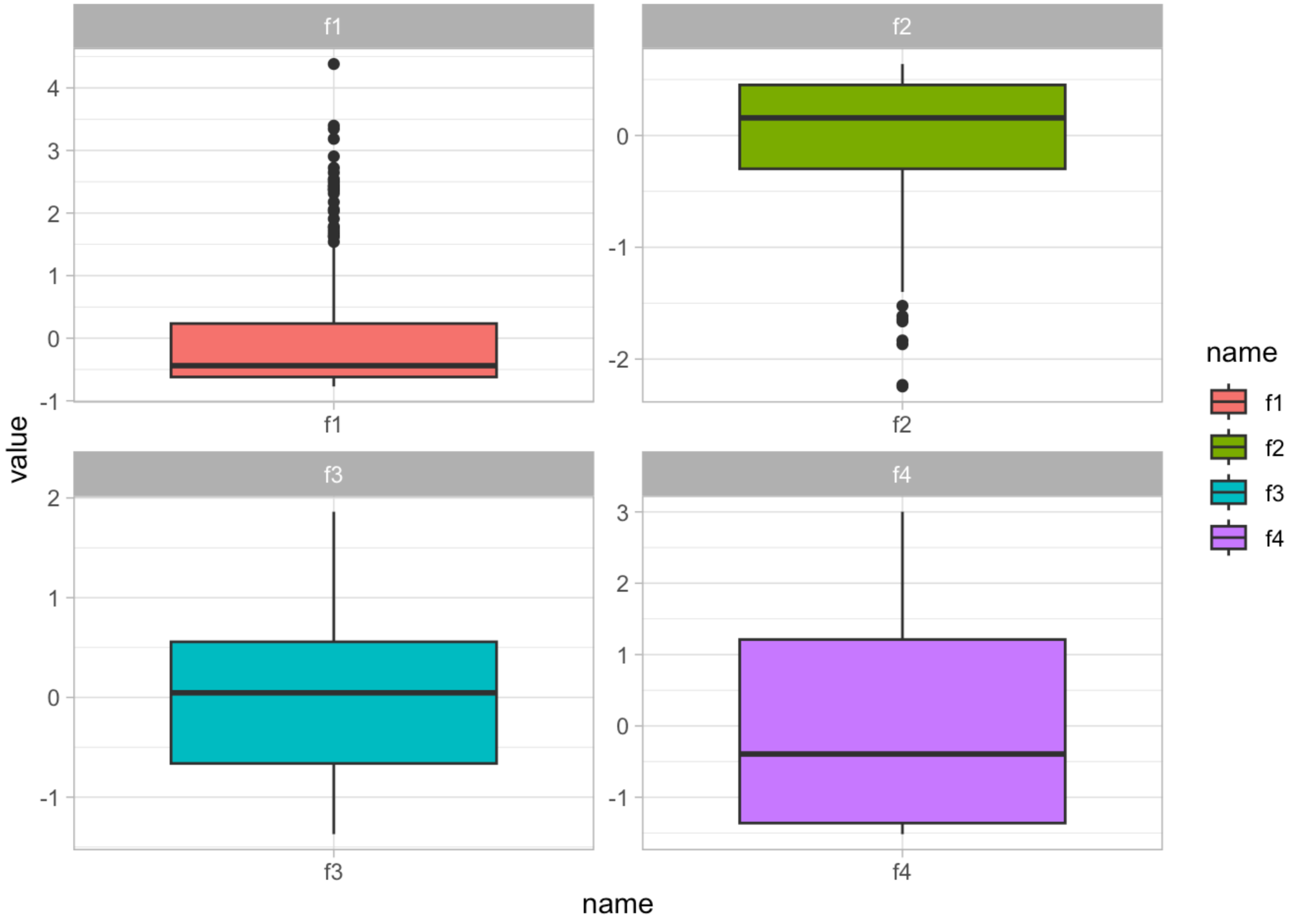


```
### Gender vs f1----f4
## testing gender and f1-f4
table(data$Gender)
```

```
##
##   1   2
## 181 112
```

```r
t1 <- t.test(f1 ~ Gender, data)
t2 <- t.test(f2 ~ Gender, data)
t3 <- t.test(f3 ~ Gender, data)
t4 <- t.test(f4 ~ Gender, data)
tab <- map_df(list(t1, t2, t3, t4), tidy)
tab
```

```
## # A tibble: 4 × 10
##   estimate estimate1 estimate2 statistic  p.value parameter conf.low conf.high
##      <dbl>     <dbl>     <dbl>     <dbl>    <dbl>     <dbl>    <dbl>     <dbl>
## 1   0.0839   0.0321   -0.0518      0.784 0.434         270.   -0.127    0.295
## 2   0.0106   0.00404  -0.00653     0.159 0.874         271.   -0.120    0.142
## 3  -0.117   -0.0447    0.0722     -1.47  0.143         269.   -0.273    0.0397
## 4   0.599    0.229    -0.370       3.80  0.000181      251.    0.289    0.909
## # ℹ 2 more variables: method <chr>, alternative <chr>
```

```r
## Gender vs f1----f4
## testing gender and f1-f4
table(data$Gender)
```

```
##
##   1   2
## 181 112
```

```r
t1 <- t.test(f1 ~ Gender, data)
t2 <- t.test(f2 ~ Gender, data)
t3 <- t.test(f3 ~ Gender, data)
t4 <- t.test(f4 ~ Gender, data)
tab <- map_df(list(t1, t2, t3, t4), tidy)
tab
```

```
## # A tibble: 4 × 10
##   estimate estimate1 estimate2 statistic  p.value parameter conf.low conf.high
##      <dbl>     <dbl>     <dbl>     <dbl>    <dbl>     <dbl>    <dbl>     <dbl>
## 1   0.0839   0.0321   -0.0518      0.784 0.434         270.   -0.127    0.295
## 2   0.0106   0.00404  -0.00653     0.159 0.874         271.   -0.120    0.142
## 3  -0.117   -0.0447    0.0722     -1.47  0.143         269.   -0.273    0.0397
## 4   0.599    0.229    -0.370       3.80  0.000181      251.    0.289    0.909
## # ℹ 2 more variables: method <chr>, alternative <chr>
```

```
data <- data %>%
  mutate(Gender = recode_factor(Gender, `1` = "Male",
                      `2` = "Female"))

p1 <- ggplot(data, aes(Gender, f1)) + geom_boxplot() + theme_light()
p2 <- ggplot(data, aes(Gender, f2)) + geom_boxplot() + theme_light()
p3 <- ggplot(data, aes(Gender, f3)) + geom_boxplot() + theme_light()
p4 <- ggplot(data, aes(Gender, f4)) + geom_boxplot() + theme_light()

ggpubr::ggarrange(p1, p2, p3, p4)
```

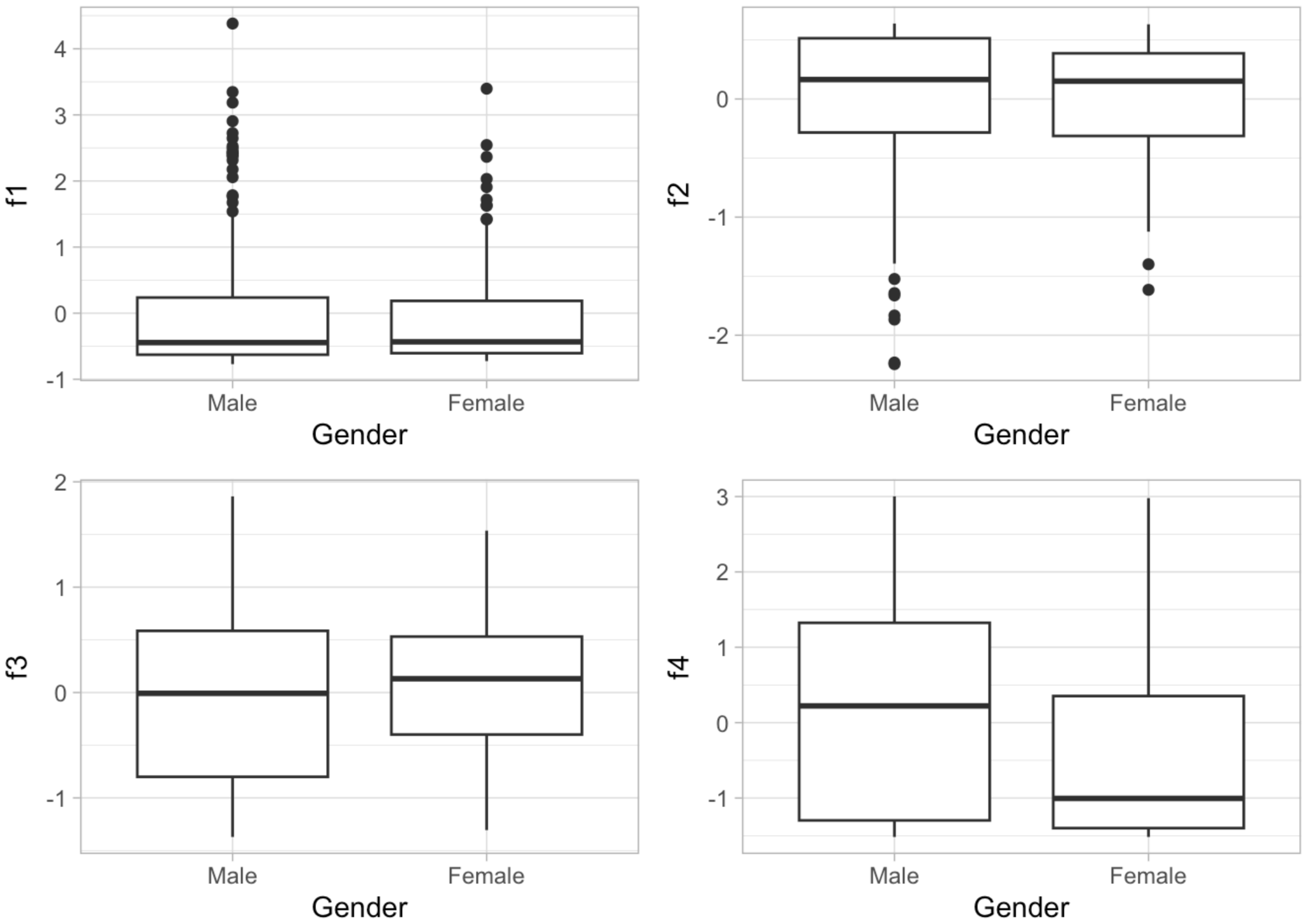


```
### Age vs f1----f4
## testing age and f1-f4

t1 <- lm(f1 ~ Age, data)
t2 <- lm(f2 ~ Age, data)
t3 <- lm(f3 ~ Age, data)
t4 <- lm(f4 ~ Age, data)
tab <- map_df(list(t1, t2, t3, t4), tidy)
tab
```

```
## # A tibble: 8 × 5
##   term        estimate std.error statistic p.value
##   <chr>          <dbl>     <dbl>     <dbl>   <dbl>
## 1 (Intercept)  0.310     0.117        2.66 0.00832
## 2 Age         -0.0131    0.00437     -2.99 0.00298
## 3 (Intercept) -0.187     0.0727      -2.57 0.0106
## 4 Age          0.00789   0.00272      2.90 0.00402
## 5 (Intercept)  0.184     0.0872       2.11 0.0358
## 6 Age         -0.00775   0.00326     -2.38 0.0181
## 7 (Intercept) -0.294     0.172       -1.71 0.0889
## 8 Age          0.0124    0.00645      1.92 0.0554
```

```
p1 <- ggplot(data, aes(Age, f1)) + geom_point() + theme_light()
p2 <- ggplot(data, aes(Age, f2)) + geom_point() + theme_light()
p3 <- ggplot(data, aes(Age, f3)) + geom_point() + theme_light()
p4 <- ggplot(data, aes(Age, f4)) + geom_point() + theme_light()

ggpubr::ggarrange(p1, p2, p3, p4)
```

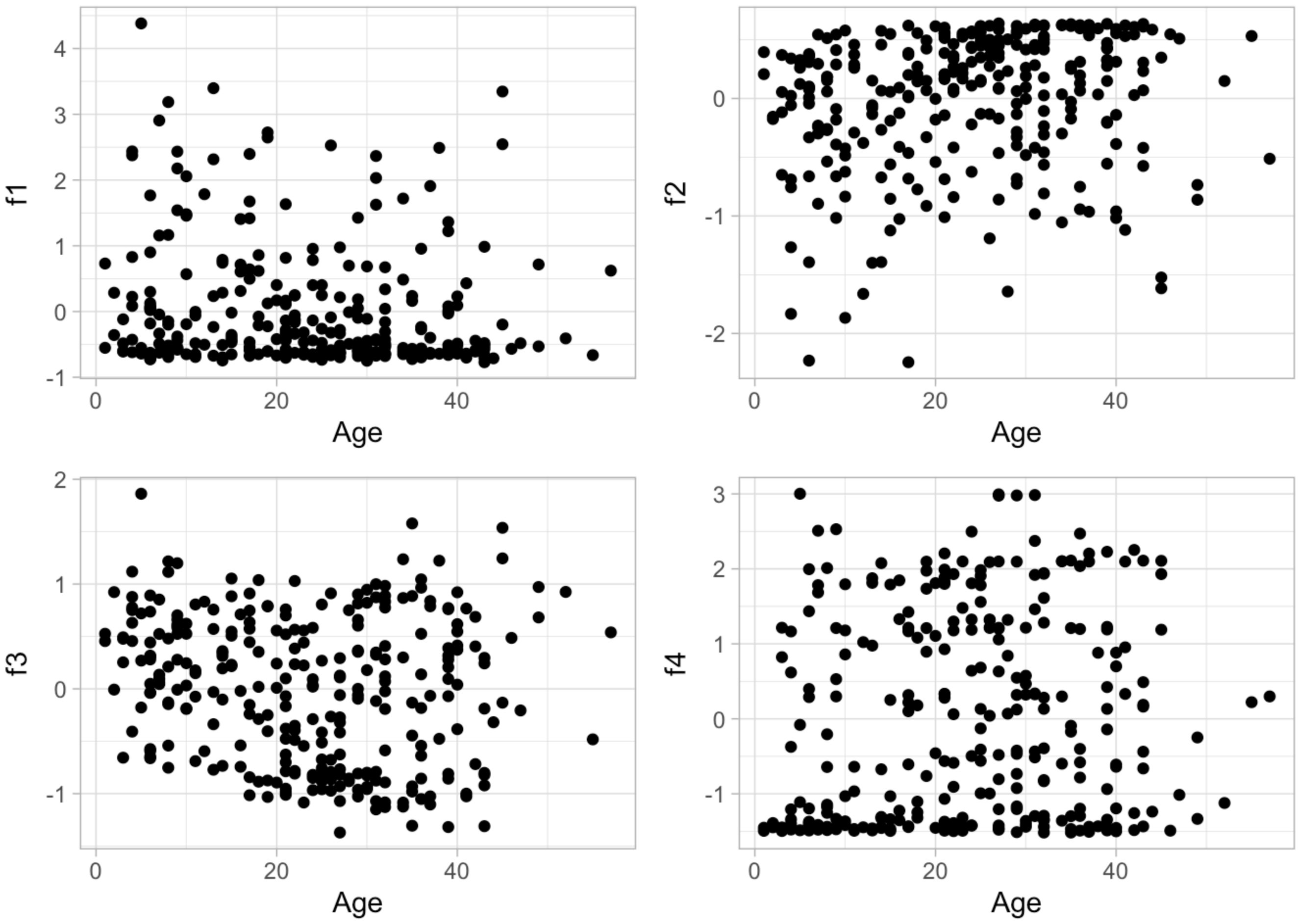


```
### lvl_competitiveness vs f1----f4
## testing lvl_competitiveness and f1-f4

t1 <- anova(lm(f1 ~ Lvl_Competitvness, data))
t2 <- anova(lm(f2 ~ Lvl_Competitvness, data))
t3 <- anova(lm(f3 ~ Lvl_Competitvness, data))
t4 <- anova(lm(f4 ~ Lvl_Competitvness, data))
tab <- map_df(list(t1, t2, t3, t4), tidy)
tab
```

```
## # A tibble: 8 × 6
##   term                 df  sumsq meansq statistic      p.value
##   <chr>             <int>  <dbl>  <dbl>     <dbl>        <dbl>
## 1 Lvl_Competitvness     1   9.95  9.95      11.8   0.000667
## 2 Residuals           291 245.    0.841     NA    NA
## 3 Lvl_Competitvness     1   8.47  8.47      27.4   0.000000320
## 4 Residuals           291  90.0   0.309     NA    NA
## 5 Lvl_Competitvness     1   7.46  7.46      16.3   0.0000685
## 6 Residuals           291 133.    0.457     NA    NA
## 7 Lvl_Competitvness     1   3.88  3.88       2.08  0.150
## 8 Residuals           291 542.    1.86      NA    NA
```

```
data <- data %>%
  mutate(Lvl_Competitvness = recode_factor(Lvl_Competitvness,
                                `1` = "Provincial",
                                `2` = "Regional",
                                `3` = "National",
                                `4` = "International"))



p1 <- ggplot(data, aes(Lvl_Competitvness, f1)) + geom_boxplot() + theme_light()
p2 <- ggplot(data, aes(Lvl_Competitvness, f2)) + geom_boxplot() + theme_light()
p3 <- ggplot(data, aes(Lvl_Competitvness, f3)) + geom_boxplot() + theme_light()
p4 <- ggplot(data, aes(Lvl_Competitvness, f4)) + geom_boxplot() + theme_light()

ggpubr::ggarrange(p1, p2, p3, p4)
```

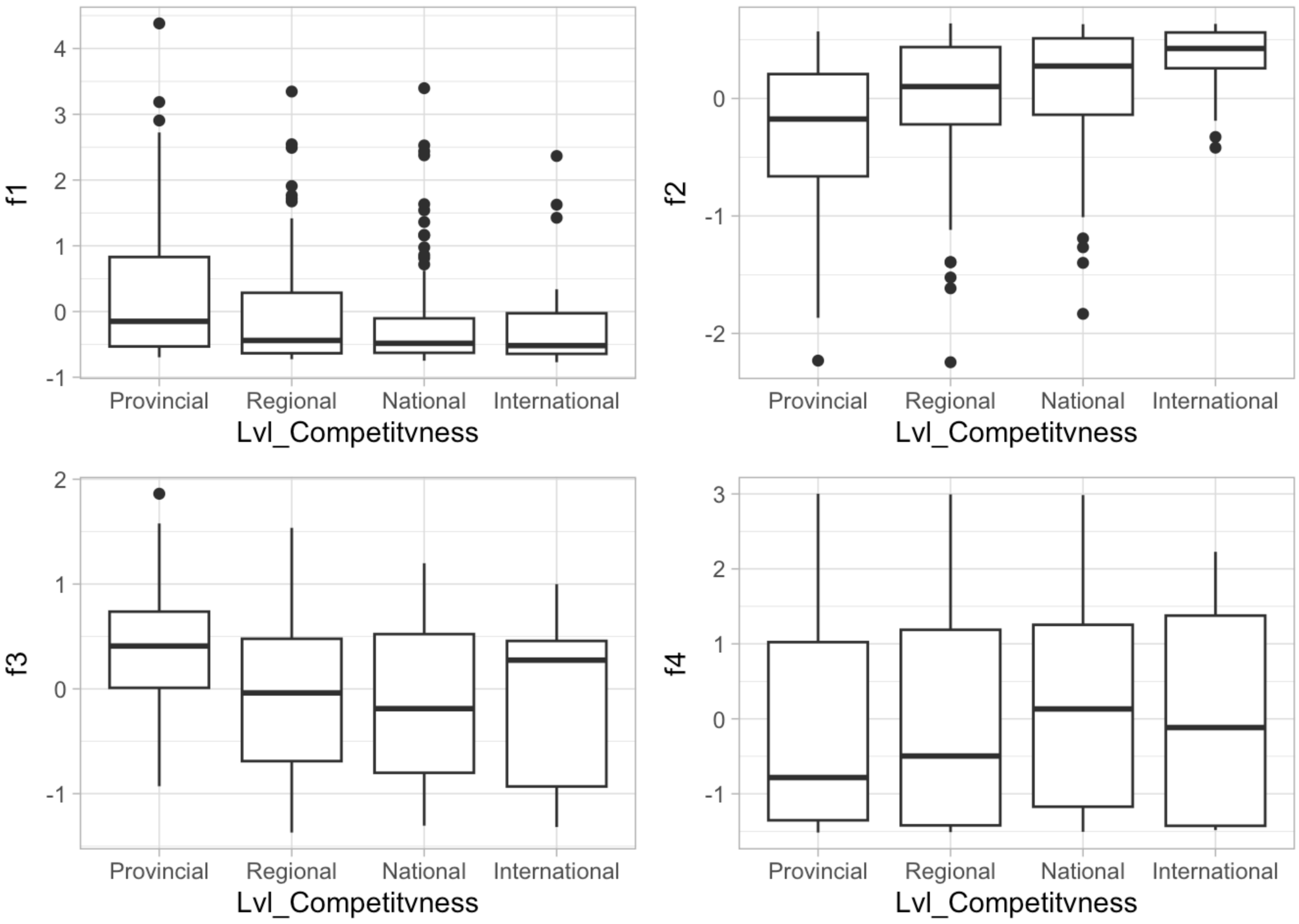


```
table(data$Lvl_Competitvness, data$Gender)
```

```
##
##                  Male Female
##   Provincial      48     17
##   Regional        57     44
##   National        63     44
##   International   13      7
```
